# A Minimally Replicative Vaccine Protects Vaccinated Piglets Against Challenge With the Porcine Epidemic Diarrhea Virus

**DOI:** 10.3389/fvets.2019.00347

**Published:** 2019-10-22

**Authors:** Gagandeep Singh, Pankaj Singh, Angela Pillatzki, Eric Nelson, Brett Webb, Steven Dillberger-Lawson, Sheela Ramamoorthy

**Affiliations:** ^1^Department of Microbiological Sciences, North Dakota State University, Fargo, ND, United States; ^2^Animal Disease Research and Diagnostic Laboratory, South Dakota State University, Brookings, SD, United States; ^3^Veterinary Diagnostic Laboratory, North Dakota State University, Fargo, ND, United States

**Keywords:** vaccine, porcine epidemic diarrhea virus, PEDV, antibody, spike

## Abstract

Porcine epidemic diarrhea virus (PEDV), is an economically important enteric coronavirus, with over a 90% mortality rate in neonatal piglets. The virus emerged in the US in 2013, resulting in severe production losses. Effective vaccine development against PEDV is a challenge. Inactivated vaccines are of questionable efficacy. Attenuated vaccines, while more effective, require a relatively long lead development time, are associated with safety concerns and are also unable to prevent new field outbreaks. To combine the safety and efficacy advantages of inactivated and attenuated PEDV vaccines, respectively, in this study, we tested the hypothesis that subjecting PEDV virions to heat treatment at 44°C for 10 min to reversibly unfold structural proteins, followed by exposure to RNAse to fragment the genome, would result in a vaccine preparation with intact viral structure/antigenicity but highly diminished replicative abilities. We expected the vaccine to be both safe and effective in a piglet challenge model. Following the heat and RNAse treatment, PEDV virions had an intact electron microscopic ultrastructure and were amplified only in the 3rd passage in Vero cells, indicating that diminished replication was achieved *in vitro*. Strong PEDV spike-protein specific and virus neutralizing antibody responses were elicited in vaccinated piglets. Upon challenge, all vaccinated pigs were protected against fecal viral shedding and intestinal pathology, while the unvaccinated controls were not. The vaccine virus was not detected in the fecal matter of vaccinated pigs prior to challenge; nor did they develop intestinal lesions. Thus, the described approach has significant promise in improving current approaches for PEDV immunization.

## Introduction

Porcine epidemic diarrhea virus (PEDV) is an enteric coronavirus which causes diarrhea, vomiting, severe dehydration, and death in pigs. Neonatal pigs are particularly susceptible, with mortality rates that can be as high as 90–100%. In older pigs, manifestation of the disease is milder but growth and production parameters are affected (1, 2). Classical strains of PEDV (G1 strains) were first detected in the UK in 1971, and spread to Asia and Europe. More recently, highly virulent strains (G2 strains) which emerged in China have spread to other countries, with the first case in the US being recorded in 2013 (1, 3, 4). It is estimated that the outbreak resulted in the losses of $0.9–1.8 billion and the death of 7 million pigs (5, 6). The availability of effective vaccines and the practice of stringent biosecurity measures are critical for the prevention of PEDV. However, the development of effective vaccines has been complicated by frequent viral evolution and the fact that PED is most severe in immunologically naïve neonates. Effective and safe vaccines development was also challenging because active vaccine replication in the gut is required to induce good and lasting mucosal immunity.

Both attenuated and inactivated PEDV vaccines have been routinely used in Asian countries for several years. Vaccination of sows prior to farrowing induces lactogenic immunity which is transferred to neonatal piglets via colostrum. Inactivated vaccines are very safe but have a low duration of immunity and appear to produce a predominantly Th2 type immune response (7). Attenuated vaccines, produced by serially passaging field strains between 83 and 100 passages, are more effective against homologous strains but have a long lead development time and have been associated with safety concerns of recombination with field strains (2). Regardless of the type of vaccine used, viremia and transmission of PEDV is not prevented in vaccinated animals. Outbreaks in vaccinated herds and the periodical emergence of new, highly pathogenic strains are not uncommon in countries were vaccines have been routinely used for many years (2, 7–9).

In North America, a S-protein based subunit vaccine (iPED plus, Harris Vaccines Inc.) and inactivated vaccines produced by Zoetis and VIDO-Intervac were conditionally licensed. However, their efficacy has also been questioned by independent studies, as vaccination of PEDV naïve sows did not result in strong protection in neonatal piglets (8, 10, 11). As the strong need for effective PEDV vaccines remains unmet, the practice of feeding back minced intestines from infected piglets to sows, in an attempt to induce more effective immunity against PEDV, is common in the field (8, 10, 11). The use of autogenous vaccines, where a custom inactivated vaccine tailored to each herd is prepared using a sample provided from the production unit, is also practiced (8, 12, 13). Both the feedback and autogenous vaccine approaches are, once again, associated with significant safety and efficacy issues but natural or intentional exposure of pigs of all ages to PEDV provides stronger homologous and partial heterologous protection (2, 8). Further, vaccination of naïve animals is less effective than vaccination of previously exposed pigs, indicating that current vaccines are less effective than natural infection at priming the immune response but can effectively boost the memory response (14). It is established that the viral spike protein is a critical protective antigen, as anti-spike protein-specific serum IgG levels correlate well with protection against PEDV and virus neutralizing responses (15). However, the S-protein based subunit vaccine (iPED plus, Harris Vaccines Inc.) is of questionable efficacy, indicating that other viral components could contribute to protection.

Based on the above, we hypothesized that development of a process whereby the structural integrity of the virus was maintained but viral replication was highly diminished but not abrogated, would result in a vaccine with the combined advantages of inactivated and attenuated vaccines, namely, high safety and efficacy margins. Previously published data shows that the SARS coronavirus capsid is metastable and can be reversibly denatured by changes in temperature or pH, with unfolding commencing at 35°C and complete denaturation occurring at 55°C (16). Hence, in this study, our vaccine development approach consisted of exposing PEDV virions to 44°C to unfold the capsid, followed by fragmentation or digestion of the genome with RNAse to diminish viral replication and subsequent refolding of the capsid at 25°C. Gamma-irradiated PEDV virions were used as an inactivated control vaccine (17). The objective of this study was to evaluate the heat and RNAse treated PEDV vaccine for its safety, immunogenicity and ability to reduce viremia in a weanling piglet model, with the ultimate goal of developing a process which can potentially reduce lead vaccine development time, is safe and be easily applied to newly emerging strains.

## Materials and Methods

### Cells and Viruses

Porcine epidemic diarrhea virus (PEDV) strain PEDV CO2013 [National Veterinary Services Laboratory (NVSL), Ames, IA] was cultured at a multiplicity index (MOI) of 0.1 using Vero cells in the presence of trypsin as previously described (18, 19). The stock virus was titrated three times to obtain the mean 50% tissue culture infectious dose [TCID_50_] using the Spearman and Karber formula (20) and stored in aliquots at −80°C until further use.

### Vaccine Preparation

To optimize the temperature, time of incubation, and dose of RNAse treatment, the virus stock was resuspended to 1 × 10^5^ TCID_50/_/ml in media (pH 7.2). Diluted virus culture was exposed to temperatures ranging from 37 to 60°C for 10 min for unfolding, followed by incubation at 25°C for 30 min for refolding, and then moved to 4°C for 1 h, as previously described for the SARS coronavirus (16). Cultures were visualized by electron microscopy to ensure structural integrity. A temperature of 44°C for 10 min was selected for unfolding. Similarly, to fragment the genomic RNA, varying combinations of concentrations of RNAse A (Ameresco) and RNAse T (Thermo Scientific) were tested by adding them to the unfolded virus cultures, followed by incubation for 5, 4, 3, or 2 h at 44°C. Treated cultures were then exposed to 25°C for 30 min for refolding and cooled down on ice for 1 h. The final optimized protocol consisted of exposing the virus culture, resuspended to 10^5^ TCID_50_/ml, to 44°C for 10 min, followed by 0.1 mg/ml of RNase A and 1 μl/ml of RNase T1 (equivalent to 10 units/ml RNase A or 1,000 units/ml of RNAse T1), incubation at 44°C for 4 h, exposure to 25°C for 30 min and cooling down on ice for 1 h before storage at −80°C for further testing. The final process was tested 3 times to ensure reproducibility.

To prepare the inactivated control vaccine, 1 × 10^5^ TCID_50/_/ml of PEDV was irradiated in a Cesium-137 source gamma (γ) irradiator at time points of 8 h to 24 h at 753 rad/min. An effective dose of 24 h (1,084,320 rad), was used to prepare the irradiated vaccine, after validation as described above.

### Viral Amplification Test

To determine the effect of the treatment on viability, the treated virus and an untreated control were serially passaged 3 times in Vero cells as described above. After each passage, flasks were subject to three freeze-thaw cycles. The culture obtained was centrifuged at 10,000 × g for 10 min 4°C to remove debris. One ml of the supernatant was used to infect Vero cell monolayers and also infect 8 well chamber slides (Nunc) to visualize viral replication by an indirect immunofluorescence assay (IFA) as described below.

### Immunofluorescence Assay

Visualization of viral replication in treated and untreated cultures was achieved using an indirect immunofluorescence assay (IFA), performed essentially as described previously (18, 19). Cultured and fixed cells were stained with polyclonal swine anti-PEDV sera (NVSL) and examined with a fluorescent microscope for green cytoplasmic fluorescence characteristic of RNA viral replication.

### Electron Microscopy

**Figure 1:**
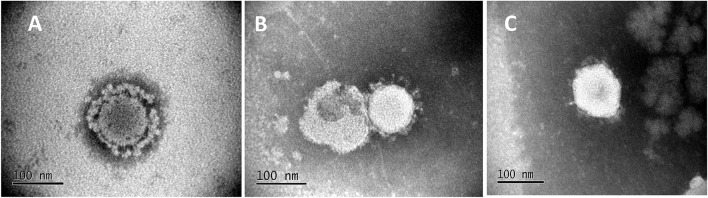
Electron Micrographs of untreated and treated PEDV. Micrographs show the characteristic corona-like structure formed by the immunogenic spike protein embedded in the virus envelop of the icosahedral virus particle. **(A)** Untreated PEDV, **(B)** Heat and RNAse treated PEDV, **(C)** Irradiated PEDV.

To visualize structure, treated, and untreated viral cultures were negatively stained by standard methods (21). Stained grids were examined with a JEOL JEM-100CX II transmission electron microscope (Figure 1).

### Deep Sequencing of Treated PEDV Virions

Possible genetic differences between untreated and treated vaccine virions were assessed by deep sequencing. Heat and RNAse treated and untreated viral particles were purified from infected cells by ultra-centrifugation at 100,000 × g for 2.50 h and re-suspended in PBS. Unpackaged RNA and DNA were removed by a RNase and DNase cocktail containing 20 units of RNase One (Promega), 20 units Benzonase (Novagen), and 14 units of turbo DNase (Ambion) incubated in 1X buffer (Ambion) for 37°C for 1.5 h. Viral RNA was then isolated by using the Qiamp Viral RNA isolation kit (Qiagen) according to the manufacturer's protocol.

Purified viral RNA was deep sequenced by a commercial vendor (BGI Genomic). The cDNA library was prepared using TruSeq library construction kit (Illumina Inc., USA) with random hexamer primers. The prepared cDNA library was then sequenced using HiSeq 4000 PE100 platform (Illumina Inc., USA) and raw reads (100 bp) were obtained. The resultant sequences reads were analyzed by BGI Genomic, Philadelphia, PA. The raw reads were filtered out using SOAPnuke to get “Clean reads” by removing the reads with adaptors, reads with more than 5% of unknown bases (N), and low-quality reads (22). Clean reads were mapped to reference PEDV genome (GenBank: KF267450.1) using HISAT (Hierarchical Indexing for Spliced Alignment of Transcripts) (23). The genome mapping results further analyzed using the Genome Analysis Toolkit (GATK) to call single nucleotide polymorphism (SNP) and INDEL (insertion and deletion of bases) (24). Only SNPs with a quality score above the threshold (Qpred > 20) and with a SNP frequency of over 85% were included in assembling the consensus sequences. The consensus sequences of the treated and untreated samples were compared by alignment with Clustal Omega (25) to obtain changes which could be attributed to the treatment. Detected changes were annotated to include the locations and proteins affected (Table 1). Clean reads were mapped to the reference genome using BOWTIE2 to detect differentially expressed genes. Gene expression levels were calculated with RSEM version 1.2.12 (26). Differentially expressed genes were identified by the possionDis, EBSeq software for samples without replicates (27).

**Table 1:** Microscopic lesion scores.

**Group**	**Mean microscopic lesion score^%^ (No of positive animals/total animals)**	**Mean IHC score ^&^(No. of positive animals/total animals)**	**Total mean histology score (No. of positive animals/total animals)**	Mean fecal score^*^	**Total necropsy score^#^**
**VACCINE EFFICACY**					
Unvaccinated	2.67 ± 1.89 (4/6)	1.5 ± 1.11 (4/6)	4.16 ± 3.25 (4/6)	2.50 ± 1.22 (5/6)	6.66 ± 3.14 (6/6)
RNase + Heat treated PEDV/Challenged	0 (0/6)	0 (0/6)	0 (0/6) (*p* = 0.03^@^)	0.50 ± 1.22 (1/6) (*p* = 0.03^@^)	0.50 ± 1.22 (1/6) (*p* = 0.004^@^)
Irradiated PEDV/Challenged	4.33 ± 3.35 (4/6)	3.0 ± 1.90 (5/6)	7.33 ± 5.49 (4/6) (*p* = 0.168)	0.50 ± 1.22 (1/6) (*p* = 0.03^@^)	7.83 ± 6.50 (5/6) (*p* = 0.37)
**VACCINE SAFETY**					
RNase + Heat treated PEDV/ Unchallenged	0 (0/2)	0 (0/2)	0 (0/2)	0 (0/2)	0 (0/2)
Irradiated PEDV/Unchallenged	0 (0/2)	0 (0/2)	0 (0/2)	0 (0/2)	0 (0/2)

*Total number of pigs = 8, No. of pigs sacrificed for vaccine safety assessment prior to challenge = 2, No. of pigs sacrificed at day 7 post challenge = 6*.

% *Total atrophic enteritis score for the ileum, jejunum, duodenum where 0, negative; 2, mild; 4, moderate; 6, severe; 2, sections with crypt hypertrophy*.

& *Total immunohistochemistry (IHC) for the Ileum, jejunum, duodenum where 0, negative; 2, positive; ≤ 10%, 4, positive, 11–50%; 6, positive, >50%*.

* *Fecal score at necropsy-Formed Feces = 0, Semi-formed feces = 3, Liquid feces = 6*.

# *Sum of the microscopic and fecal scores*.

@ *p < 0.05 as determined by the Mann–Whitney U-test, compared to the unvaccinated group*.

### Ethics Statement

All animal experimentation was approved by the Institutional Animal Care and Use Committee (IACUC) of S. Dakota State Universities (SDSU) (Protocol number: 15-013A). No other specific permissions were required for these activities. This study did not involve endangered or protected species.

### Swine Vaccine and Challenge

Twenty-four, 2 to 3-week-old piglets which were negative for PEDV by PCR and serology were divided into 3 groups; Group 1—unvaccinated control group (*N* = 8) (2 ml of PBS intramuscular and oral route each), Group 2—RNase and Heat treated PEDV vaccine group (PEDV-VAC) group (*N* = 8) (2 ml of 10^5^ TCID_50_/ml, intramuscular and oral route each) and Group 3—irradiated PEDV vaccine group (*N* = 8) (2 ml of 10^5^ TCID_50_/ml, intramuscular and oral route each). Piglets were boosted by the same route and dose at DPV 14 and 28. On DPV 43, small intestine, heart, liver, and spleen were collected 2 piglets from each group (*N* = 2/group) to assess vaccine safety. The remaining piglets (*N* = 6/group) were challenged orally with 10^5^ TCID_50_/ml of PEDV CO2013, as previously described (28, 29). Post-challenge, the piglets were observed daily for clinical signs of PED. All piglets were euthanized 1-week post challenge (DPC) or at DPV 49 and three sections of the small intestine (duodenum, jejunum, and ileum) were collected for histopathological (HP) and immunohistochemical (IHC) analysis. Serum was collected from all piglets on DPV 0, 14, 28, 43, and 49 to measure binding and neutralizing Ab responses. Fecal swabs were collected at DPV 7, 21, 38, and 42 from all piglets to measure shedding of the vaccine virus by RT-qPCR. Fecal swabs were collected on DPV 45 and 49 (DPC day 3 and 7) from all piglets to measure protection against shedding of the challenge virus by RT-qPCR.

### Antibody Responses to the PEDV Spike and Nucleoproteins

Spike protein-specific IgG responses in pigs were measured in duplicate by an indirect ELISA as previously described, using the PEDV S antigen or NP antigen for capture (18). The assay format was pre-validated at the Animal Disease Research and Diagnostic Laboratory (ADRDL), SDSU, using serum samples from animals of known serological status. A standardized operating procedure was followed in sample analysis. The results were calculated as sample to positive (S/P) ratios as follows: S/P = optical density (OD) of the sample—OD of buffer/OD of positive control—OD of the buffer.

### Fluorescent Focus Neutralization Assay

To assess the neutralizing antibody responses elicited by vaccination, a pre-validated fluorescent focus neutralization (FFN) assay was used as previously described (18), following the standard operating procedures of the ADRDL, SDSU. Briefly, doubling dilutions of heat inactivated sera were incubated with 100 foci forming units, incubated for 1 h and cultured on Vero cell monolayers. Plates were stained with a PEDV-specific fluorescein-labeled monoclonal antibody (SD6-29) to visualize the end point, which was defined as a 90% reduction of foci compared to the controls.

### RT-qPCR for Vaccine and Challenge Virus Shedding

Virus shedding through fecal route was assessed by a RT-qPCR performed by the NDSU Veterinary Diagnostic Laboratory, using pre-validated standard operating procedures, and a commercial PCR kit called the Swine Enteric PCR Panel (Thermo Fisher) following the manufacturer's instructions. Each pig was considered a biological replicate (*N* = 6, as 2 pigs/ group were sacrificed to assess vaccine safety prior to challenge), and each sample was assessed in duplicate. The obtained Ct-values were converted to viral copy numbers using a standard curve and log transformed for representation.

### Histology

Tissue samples, collected as described above, were fixed in neutral buffered formalin for 48 h, trimmed, processed, and embedded in paraffin. Tissues were cut into 5 μm thick sections and stained with hematoxylin and eosin (HE) or a PEDV N protein-specific monoclonal antibody (SD6-29) for immunohistochemistry (IHC) following the standard operating procedures of the ADRDL, SDSU. Scores were recorded in a blinded fashion by a board-certified veterinary pathologist. Scores to measure atrophic enteritis characteristic of PED were assigned as follows: 0 = negative, 2 = mild, 4 = moderate, 6 = severe. Sections with crypt hypertrophy were assigned an additional 2 points. Antigen detection in enterocytes by IHC was semi-quantitatively scored based on the following criteria: 0 = negative, 2 = positive, ≤ 10%, 4 = positive, 11–50%, 6 = positive, >50%. The consistency of fecal matter during necropsy was assigned scores as follows: Formed Feces = 0, Semi-formed feces = 3, Liquid feces = 6. Total scores were calculated as the mean sum of the histology and fecal scores (Table 1).

### Statistical Analysis

Significant differences between treatments were assessed by ANOVA and when significant (*p* < 0.05) *post-hoc* analysis was used to determine differences between groups. The Student's *T*-test was used for the serology and RT-qPCR data and the Mann–Whitney *U*-test for the pathology lesion scores. The mean values of replicates, standard deviation and statistical significance are represented in the Figures and tables.

## Results

### Treatment With Heat and RNAse Diminishes Viral Replication While Maintaining Structural Integrity

**Figure 2:**
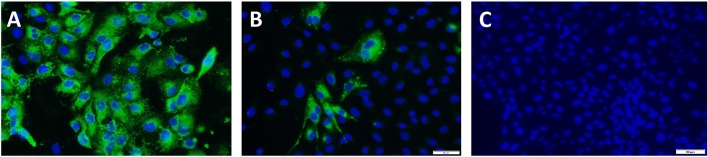
Amplification test for viral inactivation: Immunofluorescence images of vaccine viruses at the 3rd passage. Green cytoplasmic fluorescence is indicative of viral replication and blue fluorescence localizes to the nucleus of the infected Vero cells. Images were obtained by staining with a PEDV-specific polyclonal antibody. **(A)** Untreated PEDV, **(B)** Heat and RNAse treated. PEDV at the 3rd passage showing minimal replication, **(C)** Irradiated PEDV at the 3rd passage.

To achieve the targeted outcomes of maintaining structural integrity while achieving diminished viral replication, rather than complete inactivation, PEDV virus cultures were first exposed to temperatures ranging from 37 to 60°C for 10 min and visualized by electron microscopy. Intact structures were detected at all temperatures tested. However, increasing numbers of misshapen and fragmented virions were detected at 50°C and above. Cultures treated at 37 and 45°C remained viable as viral replication was visible by immunofluorescence (IFA) in infected Vero cells using a PEDV-specific antibody, without any amplification by serial passaging. Virus was detected after the 1st passage in the cultures treated at 50°C. Virus cultures treated at 55 and 60°C were not amplified even after four serial passages in Vero cells, indicating that complete inactivation occurred at these temperatures. Hence a temperature of 44°C for 10 min was chosen for reversible unfolding of the viral capsid (Figure 1B) without completely inactivating the virus. Untreated control virus culture remained structurally intact as expected (Figure 1A). Similarly, while RNAse treatment alone did not affect viability, the reduction in viral replication was proportional to the dose and time of exposure to RNase in the heat-treated virions. A dose of 10 units of RNase A and 1,000 units of RNase T with an exposure time of 4 h was chosen as optimal for the final vaccine preparation. While the untreated virus control showed robust replication (Figure 2A), following the heat and RNAse treatment protocol, viral replication was detected only in the 3rd passage in Vero cells (Figure 2B).

For the gamma (γ) irradiated, inactivated control vaccine, typical icosahedral structures were seen in electron microscopy after 23 h of exposure to radiation. However, the corona-like layer containing the protective spike antigens appeared to be damaged (Figure 1C). At this dose of radiation, the virus was not detected by the IFA with a PEDV-specific Ab at the third serial passage in cell culture (Figure 2C). Hence, a final dose of 24 h (1,084,320 rad) was selected to prepare the inactivated control vaccine.

### Vaccination of Pigs With the Heat and RNAse Treated Virions Elicits a Strong Protective Antibody (Ab) Response

**Figure 3:**
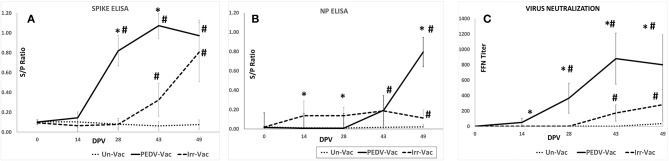
Serological responses to vaccination: **(A)** Antibody responses to the PEDV spike protein as assessed by ELISA **(B)**. Antibody responses to the PEDV nucleoprotein as assessed by ELISA **(C)**. Virus neutralizing antibody responses as assessed by a fluorescent focus neutralization (FFN) assay. X axis, Days post vaccination; Y axis, ELISA OD value expressed as a signal to positive control ratio; Line with dots, Unvaccinated controls; Solid line, Heat and RNAse treated vaccine; Dashed line, Irradiated vaccine. Mean duplicate values for 8 pigs and standard deviations are presented. *Significantly different from the unvaccinated group, ^#^Significantly different from the other vaccine group. *p < 0.05 by the Student's *T*-test.

Measurement of Ab responses against the PEDV spike and nucleocapsid proteins (NP) by ELISA (18) showed that animals vaccinated with the heat and RNAse treated virions mounted strong Ab responses against the protective PEDV spike antigen following the booster vaccinations on DPV 14 and 28 (Figures 3A,B). However, Ab responses to non-structural nucleocapsid protein (NP) remained low prior to the challenge. In pigs immunized with the irradiated vaccine, Ab responses to both viral antigens were low. The mean optical density values for the ELISAs were significantly different between the groups (Figures 3A,B).

Measurement of virus neutralizing antibodies by a fluorescent focus inhibition test (FFN) (18) showed a trend which was similar to that of the spike protein-specific Abs. Strong virus neutralizing Ab responses, were detected in animals vaccinated with the heat and RNAse treated virions but not in the pigs which received the irradiated viral vaccine. The differences between the groups was statistically significant (Figure 3C). The spike protein-specific Ab and virus neutralizing Ab levels were strongly correlated in the heat and RNAse treated PEDV vaccinated pigs, with a correlation coefficient of 95.11%. As expected, the unvaccinated control pigs remained sero-negative for the duration of the study.

### Vaccination Protects Against Fecal Viral Shedding

**Figure 4:**
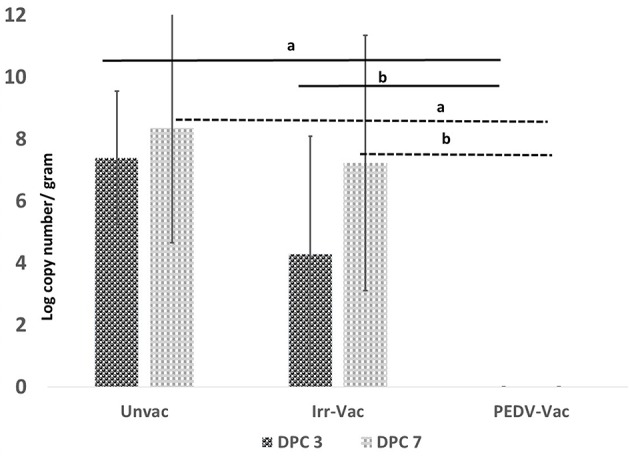
Post-challenge fecal viral loads: Viral RNA detected by a PEDV-specific RT-qPCR on day 3 and day 7 post-challenge. X axis, experimental groups *N* = 6 pigs/group (2 pigs/group were sacrificed prior to challenge to assess vaccine safety); Y axis, mean of duplicate values of viral RNA copy number per gram of fecal matter; Dark bar, Day 3 post-challenge; Light bar, Day 7 post-challenge; a, Significantly different from the unvaccinated group; b, Significantly different from the other vaccine group. *p* < 0.05 by the Student's *T*-test. Differences between the unvaccinated and irradiated vaccine group were not significant.

To assess the efficacy of the vaccine in protecting against challenge, shedding of the challenge viral RNA in fecal matter was assessed by a PEDV-specific RT-qPCR on days 0, 3, and 7 post-challenge. All experimental animals were RT-qPCR negative on day 0 post-challenge (DPC). At DPC 3 and 7, challenge viral RNA was not detected in any of the pigs vaccinated with the heat and RNAse treated PEDV vaccine (Figure 4), while 4 of the 6 pigs administered the irradiated vaccine were positive by RT-qPCR on DPC3. All 6 pigs in the irradiated vaccine group turned positive by DPC7 (Figure 4). As expected, viral RNA was detected in the fecal matter of all unvaccinated pigs on both sample collection days with titers increasing between DPC 3 and 7. While the viral RNA loads were significantly different between the two vaccine groups at both time points, there were no significant differences between the unvaccinated controls and pigs administered the irradiated vaccine at both the time points tested, indicating that the irradiated vaccine did not provide protection against viral replication and shedding in the host.

### Vaccination Protects Against Intestinal Pathology

**Figure 5:**
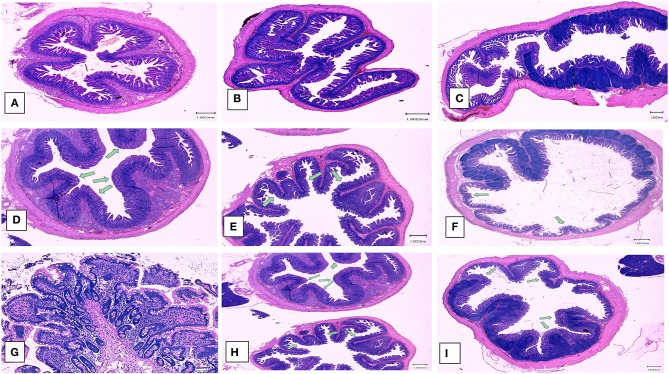
Post-challenge histopathology of small intestines. Left panel **(A,D,G)** Hematoxylin and eosin stained sections showing representative microscopic lesions (10X magnification). **(A–C)** Healthy pigs. **(A)** Duodenum, **(B)** Jejunum, **(C)** Ileum, **(D–G)** Unvaccinated, PEDV challenged pigs, **(D)** Duodenum, **(E)** Jejunum, **(F)** Ileum, **(G)** Ileum (100x). **(H,I)** Pigs vaccinated with the irradiated vaccine and challenged **(D)** Duodenum and Jejunum **(F)** Ileum. Green arrows indicate areas of villus atrophy and crypt hyperplasia.

**Figure 6:**
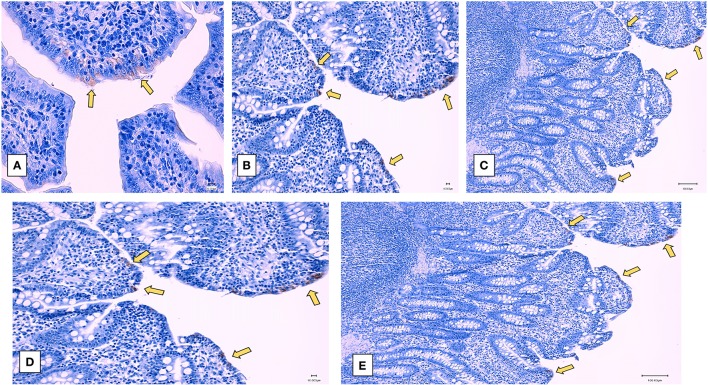
Post-challenge immunohistochemistry of small intestines—Representative immunohistochemistry images of sections stained by a PEDV-specific antibody. **(A–C)** unvaccinated challenged pigs. **(A)** Jejunum (400X), **(B)** ileum (200X), **(C)** Duodenum (100X), **(D,E)** Pigs vaccinated with the irradiated vaccine and challenged **(D)** ileum (200X) **(E)** Duodenum (100X). Yellow arrows indicate viral antigen localized to enterocytes. Pigs vaccinated with the heat and RNase treated vaccine and challenged did not show microscopic or immunohistochemistry changes (data not represented).

Examination of the intestinal tissue of the experimental animals by histology and immunohistochemistry (IHC) showed that the heat and RNAse treated PEDV vaccine completely protected vaccinated pigs against the development of microscopic lesions following challenge. Characteristic microscopic intestinal lesions of atrophic enteropathy and crypt hyperplasia were detected in the duodenum, jejunum, and ileum of animals in the control groups (Figures 5D–G). Viral antigen was also detected in the enterocytes in all three sections using a PEDV-specific monoclonal Ab-based immunohistochemistry assay (Figures 6A–E). There were no significant differences between the 3 sections, indicating the entire small intestine was affected. The total microscopic score, including the histopathology and immunohistochemistry scores was 4.16 for the unvaccinated animals and 7.33 for the pigs immunized with the irradiated vaccine and 0 for pigs administered the heat and RNAse treated vaccine. While the difference between the unvaccinated group and irradiated vaccine group was not statistically significant, the irradiated vaccine appeared to enhance intestinal pathology (Table 1). Similarly, the total necropsy scores, a sum of both the fecal and histology scores, were significantly different (*p* = 0.04) between the two vaccine groups but not between the unvaccinated group and the irradiated vaccine group (*p* = 0.37) (Table 1).

### The Experimental Vaccines Are Safe

No side effects or clinical signs of PED were observed in vaccinated pigs after either the primary or booster vaccines. Vaccine viral RNA was not detected by RT-qPCR in the fecal matter of any of the vaccinated pigs from both groups at 7 days after the primary vaccination or at 1 week after the boosters. All animals remained PCR negative until the day of challenge. Therefore, although the heat and RNase treated PEDV virions were detected by amplification after 3 serial passages in Vero cells, replication of the vaccine virus in the host appeared to be curtailed by its immune system. In the 2 pigs euthanized from each group prior to challenge, stools were fully formed at necropsy (Table 1). No microscopic lesions or viral antigen were detected in the small intestine sections, heart, spleen, and liver of the 2 animals necropsied from each group prior to challenge (Table 1). Representative images of the duodenum, jejunum, and ileum are depicted in Figure 5.

### Heat and RNAse Treatment Results in Genetic Changes

To identify possible mutations that could explain the highly effective attenuation observed, deep sequencing of heat and RNAse treated virions from infected vero cells resulted in a total of 59.42 and 24.44 MB of raw reads were obtained by RNA seq for the treated and untreated samples, respectively. Clean reads obtained after trimming were 26.94 and 19.53 GB, respectively. The Qphred20 values for the clean reads were 96.69 and 98.49 for the untreated and tread samples, respectively, indicating satisfactory quality of the data obtained. As listed in Table 2 SNPs and insertions or deletions (INDELS) were detected in the polyprotein, spike and envelope proteins (Table 2, S1 Sequence File and Supplementary Figures 1–3) of heat and RNAse treated virions, when compared to the untreated virions. In addition, insertions and deletions were detected in the S1 region for the spike protein. The N terminal signal peptide region of the spike protein had a 2 amino acid deletion and one non-synonymous change at position 355, changing the sequence from IGEN to K—N. A conservative in-frame insertion was detected at position 355 in the S1 region, changing the amino acid sequence from L----AT to LKKKGAT (Table 2 and Supplementary Figure 2).

**Table 2:** SNPs and INDELS.

**Pos**	**R**	**Un-Trt**	**Trt**	**Con-sequence**	**Residue change**	**Gene**	**AF**	**Type**
**POLY-PROTEIN**								
4,982	C	C	T	NS	S1564F	PP-NSP3	1.0	Ti
12,156	TC	TC	CG	NS	R3956G	PP-NSP9	0.99	Ti
20,203	A	A	–	Frame-shift	P6640- VGTWWYCSY. to LALGGTVAIK.	PP-NSP13	0.99	
**SPIKE PROTEIN**								
20,796	TTGGTG	TTGGTG	–	NS & Del	P55- IGEN to K–N	S-N term	1.0	
21,307	T	T	C	S	_	S-S1	1.0	Ti
21,698	–	–	AAGAAGAAAGGT	In-frame insertion, conservative	P355 L----AT to LKKKGAT	S-S1	0.86	
21,761	C	C	T	NS	L377F	S-S1	1.0	Ti
22,541	T	C	C	NS	F637L	S-S1	1.0	Ti
23,300	G	G	C	NS	G890R	S-S2	1.0	Tv
24,395	G	G	T	NS	D1211Y	S-S2	1.0	Tv
24,796	G	T	T	NS	Q1388H	S-S2	1.0	Tv
**ENVELOP PROTEIN**								
25,638	C	C	T	NS	S62F	Envelop	1.0	Ti

*Pos, position on the consensus sequence of the treated vaccine virus; R, nucleotide in the reference genome; Un-Trt, SNP in the un-treated PEDV; Trt, SNP on the treated PEDV; NS, Non-Synonymous; S, Synonymous; PP, Polyprotein; S, Spike; AF, allele frequency; Ti, transition mutation; Tv, transversion mutation*.

## Discussion

Chemical methods for inactivation of viruses have long been in use for vaccine development. While they are rapid and convenient, commonly used inactivation agents may not only affect nucleic acids but also protein structures and hence antigen presentation and vaccine efficacy. Gamma irradiation has been traditionally used to inactivate viruses. The mechanisms involved include nucleic acid degradation, destruction of covalent bonds, and release of free radicals (30). As commercial inactivated vaccines were not available at the time of testing gamma irradiation was selected as the method of choice to prepare an inactivated control vaccine for this study. Moreover, similar to the heat and RNAse treated vaccine, the virus-like-particulate structure was more likely to be maintained by gamma irradiation, while achieving complete inactivation.

Gamma irradiation had been previously used for vaccine development with varying success, depending on the pathogen (17). For example, we have previously demonstrated that a gamma irradiated vaccine against *Neospora caninum* was effective in mice (31). However, a gamma irradiated, Lassa virus vaccine failed to protect vaccinated mice (32). Although both approaches tested in this study targeted nucleic acids and preservation of structure, the protective outcomes varied significantly between the two vaccines tested. It is possible that release of free radicals during the irradiation process could have a deleterious effect on integrity of antigenic structures and antigen presentation *in vivo*. A more detailed characterization of these parameters will be the focus of future studies. Similar results for the gamma irradiated vaccine in this study, it has been shown that a dendritic cell targeted spike protein-based subunit vaccine against PEDV exacerbated intestinal pathology in vaccinated pigs, despite stimulating strong CD4^+^/CD8^+^ T cell responses (33).

While characterizing the exact physical interactions involved in the heat and RNAse treatment is not within the scope of this study, our finding that exposure of PEDV to temperatures below 50°C did not affect structure was similar to other studies showing that the SARS coronavirus structure is metastable and can be reversibly denatured by exposure to varying physical conditions such pH and temperature (16, 34). Although the heat and RNAse treated virus culture was amplified after 3 passages in cell culture (Figure 2), the absence its detection by RT-qPCR (Figure 4), or immunohistochemistry (Table 1 and Figure 5) and the lack of strong Ab responses to the non-structural NP (Figure 3), in vaccinated pigs prior to challenge indicates that active vaccine viral replication was absent in the host or was undetectable by the techniques used. Therefore, unlike other attenuated PEDV vaccines or vaccination strategies that rely on prior exposure to field strains, it is highly improbable that reversion to virulence or recombination with field strains could occur with the heat and RNAse treated vaccine.

Viral genomes that were identical to the untreated parental virus were not detected by deep sequencing of the heat and RNAse treated virus from infected Vero cells. Insertions and deletions in the spike protein, especially the S1 region, influence pathogenicity, and immunogenicity of PEDV. The core neutralizing epitope of the PEDV spike protein has been localized to amino acid positions 503–568 (35, 36). The SNPs identified in the spike protein of the vaccine virions (Table 2) did not map to these residues. While a limitation of the described method is that genetic changes induced by treatment and repair are unpredictable, repair of mutations (37) or complementation in trans of the fragmented genome could have led to detection of a fluorescent signal in the 3rd passage after treatment. Indeed, it has been shown that replication deficient genomes with deletions or mutations are produced during serial passaging of foot and mouth disease virus (FMDV) for attenuation. They are not infective by themselves, but when present in the same cell, the mutations in the genomes can complement each other in trans to produce plaques *in vitro*. When the defective-complementing virus system was used as a vaccine by Rodriguez-Calvo et al. vaccine virus replication was not detected but strong protection was elicited. This observation can be explained by vaccine virus replication in the host being limited by the requirement of coinfection of the same cell. Even if such an unlikely coinfection event were to happen despite active host innate immunity, the recombined progeny viruses were more likely to be highly attenuated than acquire virulence, thus providing an additional vaccine safety barrier *in vivo* (38). *In vivo*, the presence of the host innate immune system was likely able to effectively curtail replication, despite exposure to 10^5^ TCID_50_ of the heat and RNAse treated virus culture. More detailed studies are required to confirm these hypotheses, but they are not within the scope of this manuscript.

The importance of spike protein-specific antibodies for protection against PEDV is well-established (15). Several studies describing experimental subunit and vectored vaccines or commercial attenuated and inactivated vaccines against PEDV establish a strong correlation between spike protein-specific antibodies, virus neutralization titers and protection against infection (9, 29, 37, 39–43). Similar to these studies, strong spike-protein specific Ab responses and virus neutralizing responses were noted in the pigs immunized with the heat and RNAse treated vaccine. A commercial inactivated vaccine was able to reduce challenge viral shedding by 3–4 logs but an attenuated vaccine induced IgA responses but did not affect viral shedding (43). Testing of two attenuated PEDV strains produced by serial passage in weanling pigs showed that the passaged viruses were attenuated but were not protected against challenge viral shedding or clinical signs (29). While direct comparisons are not possible due to differences in experimental conditions, unlike the other cited studies, intestinal lesions, or challenge virus was not detected by qPCR in the heat and RNAse treated vaccine group in this study. Although boosters were incorporated in the study design to minimize risk, it is likely that they were not required to achieve adequate protection as strong spike protein specific antibody responses and virus neutralizing responses were detected after the first dose of the heat and RNAse treated vaccine, at DPV 14 (Figure 3). While cell mediated immunity was not assessed due to difficulties with transportation of cells, it is very likely that it was not compromised by the process used as the heat and RNAse treated vaccine was very effective in preventing challenge viral replication in vaccinated pigs.

While ideal for PEDV, studying vaccine efficacy in pregnant sows and neonatal pigs is expensive and procedurally tedious. Although clinical signs are less severe in older piglets (28) and virulence can vary between isolates used for challenge (44, 45), PEDV can infect and replicate well in pigs of all ages (14, 46). Hence several researchers have used weanling piglets to screen vaccine candidates for efficacy and safety (9, 13, 29, 43, 47–52). This approach can help reduce animal use and cost if the candidates fall short of expectations. Several swine bioassay studies in growing piglets have reported that peak PEDV replication occurs between DPI 3 and DPI 7 after which viral loads decrease (28, 43, 47, 48). Similar patterns of infectivity were observed in this study, as the uninfected control pigs had a mean fecal viral RNA load of 8.35 log copy numbers at DPI 7 (Figure 4) developed microscopic lesions, but not severe clinical signs. In comparison to the untreated control and irradiated vaccine groups, no fecal viral shedding or intestinal pathology was detected in the pigs immunized with the heat and RNase treated vaccine, indicating that vaccine induced immunity was highly effective against PEDV challenge, within the limits of this weanling pig study model.

The primary advantages of this innovative approach are safety, efficacy, convenience and a short development time. As the method can be easily adapted to newly evolving strains, provided they are readily cultured, this approach is very relevant to current field immunization practices of feedback exposure and autogenous vaccination. Our future goals include testing the heat and RNase treated vaccine in pregnant sows, and improving oral and respiratory mucosal vaccine delivery systems to target improved protection.

## Data Availability Statement

The datasets generated for this study are available on request to the corresponding author.

## Ethics Statement

The animal study was reviewed and approved by Institutional Animal Care and Use Committee (IACUC) of S. Dakota State Universities (SDSU) (Protocol number- 15-013A).

## Author Contributions

GS, PS, and SD-L: data collection, analysis, and manuscript editing. AP, EN, and BW: data collection and manuscript editing. SR: conception, funding, and manuscript preparation and editing.

### Conflict of Interest

The authors declare that the research was conducted in the absence of any commercial or financial relationships that could be construed as a potential conflict of interest. The described technology is covered by a provisional U.S. Patent Application (Serial No. 15/906,685).
