# Supplementary material for: A Minimally Replicative Vaccine Protects Vaccinated Piglets Against Challenge With the Porcine Epidemic Diarrhea Virus
Source: Front Vet Sci. 2019 Oct 22;6:347. doi: 10.3389/fvets.2019.00347 (PMC6817509; doi:10.3389/fvets.2019.00347)
Supplement: Supplementary file 1 [file Data_Sheet_1.docx]

**Supplementary Material**

**S1 sequence file-** Assembled consensus sequence of the recovered heat and RNAse treated vaccine virus (Gen Bank Accession submission under process)

Source 1..28043

/organism="Porcine epidemic diarrhea virus"

/mol_type="genomic RNA"

/isolate="Heat and RNAase treated Vaccine Consensus Sequence"

[CDS](https://www.ncbi.nlm.nih.gov/nuccore/KF267450.1?location=292:12600,12600:20237) join(292..12600,12600..20237)

/ribosomal_slippage

/note="1a polyprotein and 1b polyprotein; ORF1ab"

/codon_start=1

/product="polyprotein"

/translation="MASNHVTLAFANDAEISAFGFCTASEAVSYYSEAAASGFMQCRFVSFDLADTVEGLLPEDYVMVVVGTTKLSAYVDTFGSRPKNICGWLLFSNCNYFLEELELTFGRRGGNIVPVDQYMCGADGKPVLQESEWEYTDFFADSEDGQLNIAGITYVKAWIVERSDVSYASQNLTSIKSITYCSTYEHTFPDGTAMKVARTPKIKKTVVLSEPLATIYREIGSPFVDNGSDARSIIKRPVFLHAFVKCKCGSYHWTVGDWTSYVSTCCGFKCKPVLVASCSATPGSVVVTRAGAGTGVKYYNNMFLRHVADIDGLAFWRILKVQSKDDLACSGKFLEHHEEGFTDPCYFLNDSSIATKLKFDILSGKFSDEVKQAIFAGHVVVGSALVDIVDDALGQPWFIRKLGDLASAAWEQLKAVVRGLNLLSDEVVLFGKRLSCATLSIVNGVFEFIAEVPEKLAAAVTVFVNFLNELFESACDCLKVGGKTFNKVGSYVLFDNALVKLVKAKVRGPRQAGVCEVRYTSLVIGSTTKVVSKRVENANVNLVVVDEDVTLNTTGRTVVVDGLAFFESDGFYRHLADADVVIEHPVYKSACELKPVFECDPIPDFPMPVAASVAELCVQTDLLLKNYNTPYKTYSCVVRGDKCCITCTLHFTAPSYMEAAANFVDLCTKNIGTAGFHEFYITAHEQQDLQGFVTTCCTMSGFECFMPIIPQCPAVLEEIDGGSIWRSFITGLNTMWDFCKHLKVSFGLDGIVVTVARKFKRLGALLAEMYNTYLSTVVENLVLAGVSFKYYATSVPKIVLGCCFHSVKSVLASAFQIPVQAGVEKFKVFLNCVHPVVPRVIETSFVELEETTFKPPALNGSIAIVDGFAFYYDGTLYYPTDGNSVVPICFKKKGGGDVKFSDEVSVKTIDPVYKVSLEFEFESETIMAVLNKAVGNCIKVTGGWDDVVEYINVAIEVLKDHIDVPKYYIYDEEGGTDPNLPVMVSQWPLNDDTISQDLLDVEVVTDAPVDFEGDEVDSSDPDKVADVANSEPEDDGLNVAPETNVESEVEEVAATLSFIKDTPSTVTKDPFAFDFASYGGLKVLRQSHNNCWVTSTLVQLQLLGIVDDPAMELFSAGRVGPMVRKCYESQKAILGSLGDVSACLESLTKDLHTLKITCSVVCGCGTGERIYDGCAFRMTPTLEPFPYGACAQCAQVLMHTFKSIVGTGIFCRDTTALSLDSLVVKPLCAAAFIGKDSGHYVTNFYDAAMAIDGYGRHQIKYDTLNTICVKDVNWTAPFVPDVEPVLELVVKPFYSYKNVDFYQGDFSDLVKLPCDFVVNAANENLSHGGGIAKAIDVYTKGMLQKCSNDYIKAHGPIKVGRGVMLEALGLKVFNVVGPRKGKHAPELLVKAYKSVFANSGVALTPLISVGIFSVPLEESLSAFLACVGDRHCKCFCYSDKEREAIINYMDGLVDAIFKDALVDTTPVQEDVQQVSQKPVLPNFEPFRIEGAHAFYECNPEGLMSLGADKLVLFTNSNLDFCSVGKCLNNVTGGALLEAINVFKKSNKTVPAGNCVTFECADMIFITMVVLPSDGDANYDKNYARAVVKVSKLKGKLLLAVGDAMLYSKLSHLSVLGFVSTPDDVERFYANKSVVIKVTEDTRSVKTVKVESTVTYGQQIGPCLVNDTVVTDNKPVVADVVAKVVPSANWDSHYGFDKAGEFHMLDHTGFAFPSEVVNGRRVLKTTDNNCWVNVTCLQLQFARFRFKSAGLQAMWESYCTGDVAMFVHWLYWLTGVDKGQPSDSENALNMLSKYIVPAGSVTIERVTHDGCCCSKRVVTAPVVNASVLKLGVEDGLCPHGLNYIDKVVVVKGTTIVVNVGKPVVAPSHLFLKGVSYTTFLDNGNGVAGHYTVFDHDTGMVHDGDVFVPGDLNVSPVTNVVVSEQTAVVIKDPVKKVELDATKLLDTMNYASERFFSFGDFMSRNLITVFLYILSILGLCFRAFRKRDVKVLAGVPQRTGIILRKSVRYNAKALGVFFKLKLYWFKVLGKFSLGIYALYALLFMTIRFTPIGGPVCDDVVAGYANSSFDKNEYCNSVICKVCLYGYQELSDFSHTQVVWQHLRDPLIGNVMPFFYLAFLAIFGGVYVKAITLYFIFQYLNILGVFLGLQQSIWFLQLVPFDVFGDEIVVFFIVTRVLMFLKHVFLGCDKASCVACSKSARLKRVPVQTIFQGTSKSFYVHANGGSKFCKKHNFFCLNCDSYGPGCTFINDVIATEVGNVVKLNVQPTGPATILIDKVEFSNGFYYLYSGDTFWKYNFDITDNKYTCKESLKNCSIITDFIVFNNNGSNVNQVKNACVYFSQMLCKPVKLVDSALLASLSVDFGASLHSAFVSVLSNSFGKDLSSCNDMQDCKSTLGFDDVPLDTFNAAVAEAHRYDVLLTDMSFNNFTTSYAKPEEKLPVHDIATCMRVGAKIVNHNVLVKDSIPVVWLVRDFIALSEETRKYIIRTTKVKGITFMLTFNDCRMHTTIPTVCIANKKGAGLPSFSKVKKFFWFLCLFIVAVFFALSFFDFSTQVSSDSDYDFKYIESGQLKTFDNPLSCVHNVFSNFDQWHDAKFGFTPVNNPSCPIVVGVSDEARTVPGIPAGVYLAGKTLVFAINTIFGTSGLCFDASGVADKGACIFNSACTTLSGLGGTAVYCYKNGLVEGAKLYSELAPHSYYKMVDGNAVSLPEIISRGFGIRTIRTKAMTYCRVGQCVQSAEGVCFGADRFFVYNAESGSDFVCGTGLFTLLMNVISVFSKTVPVTVLSGQILFNCIIAFAAVAVCFLFTKFKRMFGDMSVGVFTVGACTLLNNVSYIVTQNTLGMLGYATLYFLCTKGVRYMWIWHLGFLISYILIAPWWVLMVYAFSAIFEFMPNLFKLKVSTQLFEGDKFVGSFENAAAGTFVLDMHAYERLANSISTEKLRQYASTYNKYKYYSGSASEADYRLACFAHLAKAMMDYASNHNDTLYTPPTVSYNSTLQAGLRKMAQPSGVVEKCIVRVCYGNMALNGLWLGDTVICPRHVIASSTTSTIDYDYALSVLRLHNFSISSGNVFLGVVGVTMRGALLQIKVNQNNVHTPKYTYRTVRPGESFNILACYDGSAAGVYGVNMRSNYTIRGSFINGACGSPGYNINNGTVEFCYLHQLELGSGCHVGSDLDGVMYGGYEDQPTLQVEGASSLFTENVLAFLYAALINGSTWWLSSSRIAVDRFNEWAVHNGMTTVVNTDCFSILAAKTGVDVQRLLASIQSLHKNFGGKQILGYTSLTDEFTTGEVIRQMYGVNLQSGYVSRACRNVLLVGSFLTFFWSELVSYTKFFWVNPGYVTPMFACLSLLSSLLMFTLKHKTLFFQVFLIPALIVTSCINLAFDVEVYNYLAEHFDYHVSLMGFNAQGLVNIFVCFVVTILHGTYTWRFFNTPVSSVTYVVALLTAAYNYFYASDILSCAMTLFASVTGNWFVGAVCYKAAVYMALRFPTFVAIFGDIKSVMFCYLVLGYFTCCFYGILYWFNRFFKVSVGVYDYTVSAAEFKYMVANGLRAPTGTLDSLLLSAKLIGIGGERNIKISSVQSKLTDIKCSNVVLLGCLSSMNVSANSTEWAYCVDLHNKINLCNDPEKAQEMLLALLAFFLSKNSAFGLDDLLESYFNDNSMLQSVASTYVGLPSYVIYENARQQYEDAVNNGSPPQLVKQLRHAMNVAKSEFDREASTQRKLDRMAEQAAAQMYKEARAVNRKSKVVSAMHSLLFGMLRRLDMSSVDTILNLAKDGVVPLSVIPAVSATKLNIVTSDIDSYNRIQREGCVHYAGTIWNIIDIKDNDGKVVHVKEVTAQNAESLSWPLVLGCERIVKLQNNEIIPGKLKQRSIKAEGDGIVGEGKALYNNEGGRTFMYAFISDKPDLRVVKWEFDGGCNTIELEPPRKFLVDSPNGAQIKYLYFVRNLNTLRRGAVLGYIGATVRLQAGKQTEQAINSSLLTLCAFAVDPAKTYIDAVKSGHKPVGNCVKMLANGSGNGQAVTNGVEASTNQDSYGGASVCLYCRAHVEHPSMDGFCRLKGKYVQVPLGTVDPIRFVLENDVCKVCGCWLANGCTCDRSIMQSTDYGLFKRVRGSSAARLEPCNGTDTQHVYRAFDIYNKDVACLGKFLKVNCVRLKNLDKHDAFYVVKRCTKSAMEHEQSIYSRLEKCGAVAEHDFFTWKDGRAIYGNVCRKDLTEYTMMDLCYALRNFDENNCDVLKSILIKVGACEESYFNNKVWFDPVENEDIHRVYALLGTIVSRAMLKCVKFCDAMVEQGIVGVVTLDNQDLNGDFYDFGDFTCSIKGMGIPICTSYYSYMMPVMGMTNCLASECFVKSDIFGEDFKSYDLLEYDFTEHKTALFNKYFKYWGLQYHPNCVDCSDEQCIVHCANFNTLFSTTIPITAFGPLCRKCWIDGVPLVTTAGYHFKQLGIVWNNDLNLHSSRLSINELLQFCSDPALLIASSPALVDQRTVCFSVAALGTGMTNQTVKPGHFNKEFYDFLLEQGFFSEGSELTLKHFFFAQKGDAAVKDFDYYRYNRPTVLDICQARVVYQIVQRYFDIYEGGCITAKEVVVTNLNKSAGYPLNKFGKAGLYYESLSYEEQDELYAYTKRNILPTMTQLNLKYAISGKERARTVGGVSLLSTMTTRQYHQKHLKSIVNTRGASVVIGTTKFYGGWDNMLKNLIDGVENPCLMGWDYPKCDRALPNMIRMISAMILGSKHTTCCSSTDRFFRLCNELAQVLTEVVYSNGGFYLKPGGTTSGDATTAYANSVFNIFQAVSANVNKLLSVDSNVCHNLEVKQLQRKLYECCYRSTTVDDQFVVEYYGYLRKHFSMMILSDDGVVCYNNDYASLGYVADLNAFKAVLYYQNNVFMSASKCWIEPDINKGPHEFCSQHTMQIVDKDGTYYLPYPDPSRILSAGVFVDDVVKTDAVVLLERYVSLAIDAYPLSKHENPEYKKVFYVLLDWVKHLYKTLNAGVLESFSVTLLEDSTAKFWDESFYANMYEKSAVLQSAGLCVVCGSQTVLRCGDCLRRPMLCTKCAYDHVIGTTHKFILAITPYVCCASDCGVNDVTKLYLGGLSYWCHDHKPRLAFPLCSAGNVFGLYKNSATGSPDVEDFNRIATSDWTDVSDYRLANDVKDSLRLFAAETIKAKEESVKSSYACATLHEVVGPKELLLKWEVGRPKPPLNRNSVFTCYHITKNTKFQIGEFVFEKAEYDNDAVTYKTTATTKLVPGMVFVLTSHNVQPLRAPTIANQERYSTIHKLHPAFNIPEAYSSLVPYYQLIGKQKITTIQGPPGSGKSHCVIGLGLYYPGARIVFTACSHAAVDSLCVKASTAYSNDKCSRIIPQRARVECYDGFKSNNTSAQYLFSTVNALPECNADIVVVDEVSMCTNYDLSVINQRISYRHVVYVGDPQQLPAPRVMISRGTLEPKDYNVVTQRMCALKPDVFLHKCYRCPAEIVRTVSEMVYENQFIPVHPDSKQCFKIFCKGNVQVDNGSSINRRQLDVVRMFLAKNPRWSKAVFISPYNSQNYVASRMLGLQIQTVDSSQGSEYDYVIYTQTSDTAHACNVNRFNVAITRAKKGILCIMCDRSLFDVLKFFELKLSDLQANEGCGLFKDCSRGDDLLPPSHANTFMSLADNFKTDQDLAVQIGVNGPIKYEHVISFMGFRFDINIPNHHTLFCTRDFAMRNVRGWLGFDVEGAHVVGSNVGTNVPLQLGFSNGVDFVVRPEGCVVTESGDYIKPVRARAPPGEQFAHLLPLLKRGQPWDVVRKRIVQMCSDYLANLSDILIFVLWAGGLELTTMRYFVKIGPSKSCDCGKVATCYNSALHTYCCFKHALGCDYLYNPYCIDIQQWGYKGSLSLNHHEHCNVHRNEHVASGDAIMTRCLAIHDCFVKNVDWSITYPFIGNEAVINKSGRIVQSHTMRSVLKLYNPKAIYDIGNPKGIRCAVTDAKWFCFDKNPTNSNVKTLEYDYITHGQFDGLCLFWNCNVDMYPEFSVVCRFDTRCRSPLNLEGCNGGSLYVNNHAFHTPAFDKRAFAKLKPMPFFFYDDTECDKLQDSINYVPLRASNCITKCNVGGAVCSKHCAMYHSYVNAYNTFTSAGFTIWVPTSFDTYNLWQTFSNNLQGLENIAFNVVKKGSFVGAEGELPVAVVNDKVLVRDGTVDTLVFTNKTSLPTNVAFELYAKRKVGLTPPITILRNLGVVCTSKCVIWDYEAERPLTTFTKDVCKYTDFEGDVCTLFDNSIVGSLERFSMTQNAVLMSLTAVKKLTGIKLTYGYLNGVPVNTHEDKPFTWYIYTRKNGKFEDHPDGYFTQGRTTADFSPRSDMEKDFLSMDMGLFINKYGLEDYGFEHVVYGDVSKTTLGGLHLLISQVRLACMGVLKIDEFVSSNDSTLKSCTVTYADNPSSKMVCTYMDLLLDDFVSILKSLDLGVVSKVHEVMVDCKMWRWMLWCKDHKLQTFYPQLQASEWKCGYSMPSIYKIQRMCLEPCNLYNYGAGIKLPDGIMFNVVKYTQLCQYLNSTTMCVPHHMRVLHLGAGSDKGVAPGTAVLRRWLPLDAIIVDNDSVDYVSDADYSVTGDCSTLYLSDKFDLVISDMYDGKIKSCDGENVSKEGFFPYINGVITEKLALGGTVAIK”

[CDS](https://www.ncbi.nlm.nih.gov/nuccore/KF267450.1?from=20634&to=24794) 20633..24799

/codon_start=1

/product="spike protein"

/translation=”MKSLTYFWLFLPVLSTLSLPQDVTRCSANTNFRRFFSKFNVQAPAVVVLGGYLPKNQGVNSTWYCAGQHPTASGVHGIFVSHIRGGHGFEIGISQEPFDPSGYQLYLHKATNGNTNATARLRICQFPSIKTLGPTANNDVTTGRNCLFNKAIPAHMSEHSVVGITWDNDRVTVFSDKIYYFYFKNDWSRVATKCYNSGGCAMQYVYEPTYYMLNVTSAGEDGISYQPCTANCIGYAANVFATEPNGHIPEGFSFNNWFLLSNDSTLVHGKVVSNQPLLVNCLLAIPKIYGLGQFFSFNQTIDGVCNGAAVQRAPEALRFNINDISVILAEGSIVLHTALGTNFSFVCSNSSNPHLKKKGATFAIPLGATQVPYYCFFKVDTYNSTVYKFLAVLPPTVREIVITKYGDVYVNGFGYLHLGLLDAVTINFTGHGTDDDVSGFWTIASTNFVDALIEVQGTAIQRILYCDDPVSQLKCSQVAFDLDDGFYTISSRNLLSHEQPISFVTLPSFNDHSFVNITVSASFGGHSGANLIASDTTINGFSSFCVDTRQFTISLFYNVTNSYGYVSKSQDSNCPFTLQSVNDYLSFSKFCVSTSLLASACTIDLFGYPEFGSGVKFTSLYFQFTKGELITGTPKPLEGVTDVSFMTLDVCTKYTIYGFKGEGIITLTNSSFLAGVYYTSDSGQLLAFKNVTSGAVYSVTPCSFSEQAAYVDDDIVGVISSLSSSTFNSTRELPGFFYHSNDGSNCTEPVLVYSNIGVCKSGSIGYVPSQSGQVKIAPTVTGNISIPTNFSMSIRTEYLQLYNTPVSVDCATYVCNGNSRCKQLLTQYTAACKTIESALQLSARLESVEVNSMLTISDEALQLATISSFNGDGYNFTNVLGVSVYDPASRRVVQKRSFIEDLLFNKVVTNGLGTVDEDYKRCSNGRSVADLVCAQYYSGVMVLPGVVDAEKLHMYSASLIGGMVLGGFTSAAALPFSYAVQARLNYLALQTDVLQRNQQLLAESFNSAIGNITSAFESVKEAISQTSKGLNTVAHALTKVQEVVNSQGAALTQLTVQLQHNFQAISSSIDDIYSRLDILSADAQVDRLITGRLSALNAFVAQTLTKYTEVQASRKLAQQKVNECVKSQSQRYGFCGGDGEHIFSLVQAAPQGLLFLHTVLVPSDFVDVIAIAGLCVNDEIALTLREPGLVLFTHELQNHTATEYFVSSRRMFEPRKPTVSDFVQIESCVVTYVNLTRDQLPDVIPDYIDVNKTLYEILASLPNRTGPSLPLDVFNATYLNLTGEIADLEQRSESLRNTTEELQSLIYNINNTLVDLEWLNRVETYIKWPWWVWLIIFIVLIFVVSLLVFCCISTGCCGCCGCCCACFSGCCRGPRLQPYEVFEKVHVH”

[CDS](https://www.ncbi.nlm.nih.gov/nuccore/KF267450.1?from=20634&to=24794) 24799..25473

/codon_start=1

/product="hypothetical protein"

/translation=”MFLGLFQYTIDTVVKDVSKSANLSLDAVQELELNVVPIRQASNVTGFLFTSVFIYFFALFKASSLRRNYIMLAARFAVIVLYCPLLYYCGAFLDATIICCTLIGRLCLVCFYSWRYKNALFIIFNTTTLSFLNGKAAYYDGKSIVILEGGDHYITFGNSFVAFVSSIDLYLAIRGRQEADLQLLRTVELLDGKKLYVFSQHQIVGITNAAFDSIQLDEYATISE”

CDS 25454..25684

/codon start=1

/product=”envelope protein”

/translation=”MLQLVNDNGLVVNVILWLFVLFFLLIISITFVQLVNLCFTCHRLCNSAVYTPIGRLYRVYKFYMQIDPLPSTVIDV”

CDS 25692..26372

/codon start=1

/product=”membrane protein”

translation=”MSNGSIPVDEVIQHLRNWNFTWNIILTILLVVLQYGHYKYSAFLYGVKMAILWILWPLVLALSLFDAWASFQVNWVFFAFSILMACITLMLWIMYFVNSIRLWRRTHSWWSFNPETDALLTTSVMGRQVCIPVLGAPTGVTLTLLSGTLLVEGYKVATGVQVSQLPNFVTVAKATTTIVYGRVGRSVNASSGTGWAFYVRSKHGDYSAVSNPSSVLTDSEKVLHLV”

CDS 26384..27709

/codon start=1

/product=”nucleocapsid protein”

translation=”MASVSFQDRGRKRVPLSLYAPLRVTNDKPLSKVLANNAVPTNKGNKDQQIGYWNEQIRWRMRRGERIEQPSNWHFYYLGTGPHADLRYRTRTEGVFWVAKEGAKTEPTNLGVRKASEKPIIPNFSQQLPSVVEIVEPNTPPTSRANSRSRSRGNGNNRSRSPSNNRGNNQSRGNSQNRGNNQGRGASQNRGGNNNNNNKSRNQSKNRNQSNDRGGVTSRDDLVAAVKDALKSLGIGENPDKLKQQQKPKQERSDSSGKNTPKKNKSRATSKERDLKDIPEWRRIPKGENSVAACFGPRGGFKNFGDAEFVEKGVDASGYAQIASLAPNVAALLFGGNVAVRELADSYEITYNYKMTVPKSDPNVELLVSQVDAFKTGNAKPQRKKEKKNKRETTQQLNEEAIYDDVGVPSDVTHANLEWDTAVDGGDTAVEIINEIFDTGN”

ORIGIN

TTAAAAGAGATTTTCTATCTACGGATAGTTAGCTCTTTTTCTAGACTCTTGTCTACTCAATTCAACTAAACGAAATTTTGTCCTTCCGGCCGCATGTCCATGCTGCTGGAAGCTGACGTGGAATTTCATTAGGTTTGCTTAAGTAGCCATCGCAAGTGCTGTGCTGTCCTCTAGTTCCTGGTTGGCGTTCCGTCGCCTTCTACATACTAGACAAACAGCCTTCCTCCGGTTCCGTCTGGGGGTTGTGTGGATAACTAGTTCTGTCTAGTTTGAAACCAGTAACTGTCGGCTATGGCTAGCAACCATGTTACATTGGCTTTTGCCAATGATGCAGAAATTTCAGCTTTTGGCTTTTGCACTGCTAGTGAAGCCGTCTCATACTATTCTGAGGCCGCCGCTAGTGGATTTATGCAATGCCGTTTCGTGTCCTTCGATCTCGCTGACACTGTTGAGGGATTGCTTCCCGAAGACTATGTCATGGTGGTGGTCGGCACTACCAAGCTTAGTGCGTATGTGGACACTTTTGGTAGCCGCCCCAAAAACATTTGTGGTTGGCTGTTATTTTCTAACTGTAATTACTTCCTCGAAGAGTTAGAGCTTACTTTTGGTCGTCGTGGTGGTAACATCGTGCCAGTTGACCAATACATGTGTGGCGCTGACGGTAAACCTGTTCTTCAGGAATCCGAATGGGAGTATACAGATTTCTTTGCTGACTCCGAAGACGGTCAACTCAACATTGCTGGTATCACTTATGTGAAGGCCTGGATTGTAGAGCGATCGGATGTCTCTTATGCGAGTCAGAATTTAACATCTATTAAGTCTATTACTTACTGTTCAACCTATGAGCATACTTTTCCTGATGGTACTGCCATGAAGGTTGCACGTACTCCAAAGATTAAGAAGACTGTTGTCTTGTCTGAGCCACTTGCTACTATCTACAGGGAAATTGGTTCTCCTTTTGTGGATAATGGGAGCGATGCTCGTTCTATCATTAAGAGACCAGTGTTCCTCCACGCTTTTGTTAAGTGTAAGTGTGGTAGTTATCATTGGACTGTTGGTGATTGGACTTCCTATGTCTCCACTTGCTGTGGCTTTAAGTGTAAGCCAGTCCTTGTGGCTTCATGCTCTGCTACGCCTGGTTCTGTTGTGGTTACGCGCGCTGGTGCTGGCACTGGTGTTAAGTATTACAACAACATGTTCCTGCGCCATGTGGCAGACATTGATGGGTTGGCATTCTGGCGAATTCTCAAGGTGCAGTCCAAAGACGACCTCGCTTGCTCTGGTAAATTCCTTGAACACCATGAGGAAGGTTTCACAGATCCTTGCTACTTTTTGAATGACTCGAGCATTGCTACTAAGCTCAAGTTTGACATCCTTAGTGGCAAGTTTTCTGATGAAGTCAAACAAGCTATCTTTGCTGGTCATGTTGTTGTTGGCAGCGCGCTCGTTGACATTGTTGACGATGCACTGGGACAGCCTTGGTTTATACGTAAGCTTGGTGACCTTGCAAGTGCAGCTTGGGAGCAGCTTAAGGCTGTCGTTAGAGGCCTTAACCTCCTGTCTGATGAGGTCGTGCTCTTTGGCAAAAGACTTAGCTGTGCCACTCTTAGTATCGTTAACGGTGTTTTTGAGTTCATCGCCGAAGTGCCTGAGAAGTTGGCTGCGGCTGTTACAGTTTTTGTCAACTTCTTGAATGAGCTTTTTGAGTCTGCCTGTGACTGCTTAAAGGTCGGAGGTAAAACCTTTAACAAGGTTGGCTCTTATGTTCTTTTTGACAACGCATTGGTTAAGCTTGTCAAGGCAAAAGTTCGCGGCCCACGACAGGCAGGTGTTTGTGAAGTTCGTTACACAAGCCTTGTTATTGGGAGTACTACCAAGGTGGTTTCCAAGCGCGTTGAAAATGCCAATGTGAATCTCGTCGTCGTTGACGAGGATGTGACCCTCAACACCACTGGTCGTACAGTTGTTGTTGACGGACTTGCATTCTTCGAGAGTGACGGGTTTTACAGACATCTTGCTGATGCTGACGTTGTCATTGAACATCCTGTTTATAAGTCTGCTTGTGAGCTCAAGCCAGTTTTTGAGTGTGACCCAATACCTGATTTTCCTATGCCTGTGGCCGCTAGTGTTGCAGAGCTTTGTGTGCAAACTGATCTGTTGCTTAAAAATTACAACACTCCTTATAAAACTTACAGCTGCGTTGTGAGAGGTGATAAGTGTTGTATCACTTGCACCTTACATTTCACAGCACCAAGTTATATGGAGGCTGCTGCTAATTTTGTAGACCTCTGTACCAAGAACATTGGTACTGCTGGTTTTCATGAGTTTTACATTACGGCCCATGAACAACAGGATCTGCAAGGGTTCGTAACCACTTGTTGCACGATGTCAGGTTTTGAGTGTTTTATGCCTATAATCCCACAGTGTCCAGCAGTGCTTGAAGAGATTGATGGTGGTAGCATCTGGCGGTCTTTTATCACTGGTCTTAATACAATGTGGGATTTTTGCAAGCATCTTAAAGTCAGCTTTGGACTAGATGGCATTGTTGTCACTGTAGCACGCAAATTTAAACGACTTGGTGCTCTCTTGGCAGAAATGTATAACACTTACCTTTCAACTGTGGTGGAAAACTTGGTACTGGCCGGTGTTAGCTTCAAGTATTATGCCACCAGTGTCCCAAAAATTGTTTTGGGCTGTTGTTTTCACAGTGTTAAAAGTGTTCTTGCAAGTGCCTTCCAGATTCCTGTCCAGGCAGGCGTTGAGAAGTTTAAAGTCTTCCTTAACTGTGTTCACCCTGTTGTACCACGTGTCATTGAAACTTCTTTTGTGGAATTAGAAGAGACGACATTTAAACCACCAGCACTCAATGGTAGTATTGCTATTGTTGATGGCTTTGCTTTCTATTATGATGGAACACTATACTATCCCACCGATGGTAATAGCGTTGTTCCTATCTGCTTTAAGAAGAAAGGTGGTGGTGATGTCAAATTCTCTGATGAAGTCTCTGTTAAAACCATTGACCCAGTTTATAAGGTCTCCCTTGAATTTGAGTTCGAGTCTGAGACTATTATGGCTGTGCTTAATAAGGCTGTTGGTAATTGTATCAAGGTTACAGGTGGTTGGGACGATGTTGTTGAGTATATCAATGTTGCCATTGAGGTTCTTAAAGATCACATCGATGTGCCTAAGTACTACATCTATGATGAGGAAGGTGGCACCGATCCTAATCTGCCCGTAATGGTTTCTCAGTGGCCGTTGAATGATGACACGATCTCACAGGATCTGCTTGATGTTGAAGTTGTTACTGATGCGCCAGTTGATTTCGAGGGTGATGAAGTAGACTCCTCTGACCCTGATAAGGTGGCAGACGTGGCTAACTCTGAGCCTGAGGATGACGGTCTTAATGTAGCTCCTGAAACAAATGTAGAGTCTGAAGTTGAGGAAGTTGCCGCAACCTTGTCCTTTATTAAAGATACACCTTCCACAGTTACTAAGGATCCTTTTGCTTTTGACTTTGCAAGCTATGGAGGACTTAAGGTTTTAAGACAATCTCATAACAACTGCTGGGTTACTTCTACCTTGGTGCAGCTACAATTGCTTGGCATCGTTGATGACCCTGCAATGGAGCTTTTTAGTGCTGGTAGAGTTGGTCCAATGGTTCGCAAATGCTATGAGTCACAAAAGGCTATCTTGGGATCTTTGGGTGATGTGTCGGCTTGCCTAGAGTCTCTGACTAAGGACCTACACACACTTAAGATTACCTGTTCTGTAGTCTGTGGTTGTGGTACTGGTGAACGTATCTATGATGGTTGTGCTTTTCGTATGACGCCAACTTTGGAACCGTTCCCATATGGTGCTTGTGCTCAGTGTGCTCAAGTTTTGATGCACACTTTTAAAAGTATTGTTGGCACCGGCATCTTTTGTCGAGATACTACTGCTCTCTCCTTGGATTCTTTGGTTGTAAAACCTCTTTGTGCGGCTGCTTTTATAGGCAAGGATAGTGGTCATTATGTCACTAACTTTTATGATGCTGCTATGGCTATTGATGGTTATGGTCGTCATCAGATAAAGTATGACACACTGAACACTATTTGTGTTAAAGACGTTAATTGGACAGCACCTTTTGTCCCAGACGTTGAGCCTGTATTGGAGCTTGTTGTCAAACCTTTCTATTCTTATAAGAATGTTGATTTTTACCAAGGAGATTTTAGTGACCTTGTTAAACTTCCATGTGATTTTGTTGTTAATGCTGCAAATGAGAATTTGTCTCACGGTGGCGGCATAGCAAAGGCCATTGATGTTTATACCAAGGGCATGTTGCAGAAGTGCTCGAATGATTACATTAAAGCACACGGTCCCATTAAAGTTGGACGTGGTGTCATGTTGGAGGCATTAGGTCTTAAGGTCTTTAATGTTGTTGGTCCACGTAAGGGTAAGCATGCACCTGAGCTTCTTGTTAAGGCTTATAAGTCCGTTTTTGCTAATTCAGGTGTTGCTCTTACACCTTTGATTAGTGTTGGAATTTTTAGTGTTCCTTTGGAAGAATCTTTATCTGCTTTTCTTGCATGTGTTGGTGATCGCCACTGTAAGTGCTTTTGTTATAGTGACAAAGAGCGCGAGGCGATCATTAATTACATGGATGGCTTGGTAGATGCTATTTTCAAAGATGCACTTGTTGATACTACTCCTGTCCAGGAAGATGTTCAACAAGTTTCACAAAAACCAGTTTTGCCTAATTTTGAACCTTTCAGGATTGAAGGTGCTCATGCTTTCTATGAGTGCAACCCTGAAGGTTTGATGTCATTAGGTGCTGACAAGCTGGTGTTGTTTACAAATTCCAATTTGGATTTTTGTAGCGTTGGTAAGTGTCTTAACAATGTGACTGGCGGTGCATTGCTTGAAGCCATAAATGTATTTAAAAAGAGTAACAAAACAGTGCCTGCTGGCAACTGTGTTACTTTTGAGTGTGCAGATATGATTTTTATTACTATGGTAGTATTGCCATCTGACGGTGATGCTAATTATGACAAAAATTATGCACGCGCCGTCGTCAAGGTATCTAAGCTTAAAGGCAAGTTATTGCTTGCTGTTGGTGATGCCATGTTGTATTCCAAGTTGTCCCACCTCAGCGTGTTAGGTTTCGTATCCACACCTGATGATGTGGAGCGTTTCTACGCAAATAAGAGTGTGGTTATTAAAGTTACTGAGGATACACGTAGTGTTAAGACTGTTAAAGTAGAATCCACTGTTACTTATGGACAACAAATTGGACCTTGTCTTGTTAATGACACCGTTGTCACAGACAACAAACCTGTTGTTGCTGATGTTGTAGCTAAGGTTGTACCAAGTGCTAATTGGGATTCACATTATGGTTTTGATAAGGCTGGTGAGTTCCACATGCTAGACCATACTGGGTTTGCCTTTCCTAGTGAAGTTGTTAACGGTAGGCGTGTGCTTAAAACCACAGATAATAACTGTTGGGTTAATGTTACATGTTTACAATTACAGTTTGCTAGATTTAGGTTCAAGTCAGCAGGTCTACAGGCTATGTGGGAGTCCTATTGTACTGGTGATGTTGCTATGTTTGTGCATTGGTTGTACTGGCTTACTGGTGTTGACAAAGGTCAGCCTAGTGATTCAGAAAATGCACTTAACATGTTGTCTAAGTACATTGTTCCTGCTGGTTCTGTCACTATTGAACGTGTCACGCATGACGGTTGTTGTTGTAGTAAGCGTGTTGTCACTGCACCAGTTGTGAATGCTAGCGTGTTGAAGCTTGGCGTCGAGGATGGTCTTTGTCCACATGGTCTTAACTACATTGACAAAGTTGTTGTAGTTAAAGGTACTACAATTGTTGTCAATGTTGGAAAACCTGTAGTGGCACCATCGCACCTCTTTCTTAAGGGTGTTTCCTACACAACATTCCTAGATAATGGTAACGGTGTTGCCGGCCATTATACTGTTTTTGATCATGACACTGGTATGGTGCATGATGGAGATGTTTTTGTACCAGGTGATCTCAATGTGTCTCCTGTTACAAATGTTGTCGTCTCAGAGCAGACGGCTGTTGTGATTAAAGACCCTGTGAAGAAAGTAGAGTTAGACGCTACAAAGCTGTTAGACACTATGAATTATGCATCGGAAAGATTCTTTTCCTTTGGTGATTTTATGTCACGTAATTTAATTACAGTGTTTTTGTACATCCTTAGTATTTTGGGTCTCTGTTTTAGGGCCTTTCGTAAGAGGGATGTTAAAGTTCTAGCTGGTGTACCCCAACGTACTGGTATTATATTGCGTAAAAGTGTGCGCTATAATGCAAAGGCTTTGGGTGTCTTCTTCAAGCTAAAACTTTATTGGTTCAAAGTTCTTGGTAAGTTTAGTTTGGGTATTTATGCATTGTATGCATTACTATTCATGACAATACGCTTTACACCTATAGGTGGCCCTGTTTGTGATGATGTTGTTGCTGGTTATGCTAATTCTAGTTTTGACAAGAATGAGTATTGCAACAGTGTTATTTGTAAGGTCTGTCTCTATGGGTACCAGGAACTTTCGGACTTCTCTCACACACAGGTAGTATGGCAACACCTTAGAGACCCATTAATTGGTAATGTGATGCCTTTCTTTTATTTGGCATTTCTGGCAATTTTTGGGGGTGTTTATGTAAAGGCTATTACTCTCTATTTTATTTTCCAGTATCTTAACATACTTGGTGTGTTTTTGGGCCTACAACAGTCCATTTGGTTTTTGCAGCTTGTGCCTTTTGATGTCTTTGGTGACGAGATCGTCGTCTTTTTCATCGTTACACGCGTATTGATGTTCCTTAAGCATGTTTTCCTTGGCTGCGATAAGGCATCTTGTGTGGCTTGCTCTAAGAGTGCTCGCCTTAAGCGCGTTCCTGTCCAGACTATTTTTCAGGGTACTAGCAAATCCTTCTACGTACATGCCAATGGTGGTTCTAAGTTCTGTAAGAAGCACAATTTCTTTTGTTTAAATTGTGATTCTTATGGTCCAGGCTGCACTTTTATTAATGACGTCATTGCAACTGAAGTTGGTAATGTTGTCAAACTTAATGTGCAACCGACAGGTCCTGCCACTATTCTTATTGACAAGGTTGAATTCAGTAATGGTTTTTACTATCTTTATAGTGGTGACACATTTTGGAAGTACAACTTTGACATAACAGATAACAAATACACTTGCAAAGAGTCACTTAAAAATTGTAGCATAATCACAGACTTTATTGTTTTTAACAATAATGGTTCCAATGTAAATCAGGTTAAGAATGCATGTGTGTATTTTTCACAGATGCTTTGTAAACCTGTTAAGTTAGTGGACTCAGCGTTGTTGGCCAGTTTGTCTGTTGATTTTGGTGCAAGCTTACATAGTGCTTTTGTTAGTGTGTTGTCGAATAGTTTTGGCAAAGACCTGTCAAGTTGTAATGACATGCAGGATTGCAAGAGCACATTGGGTTTTGATGATGTACCATTGGATACCTTTAATGCTGCTGTTGCTGAGGCTCATCGTTACGATGTCCTCTTGACTGACATGTCGTTCAACAATTTTACCACCAGTTATGCAAAACCAGAGGAAAAACTTCCCGTCCATGACATTGCCACGTGTATGCGTGTAGGTGCCAAGATTGTTAATCATAACGTTCTTGTCAAGGATAGTATACCTGTGGTGTGGCTTGTACGTGATTTCATTGCCCTTTCTGAAGAAACTAGGAAGTACATTATTCGTACGACTAAAGTTAAGGGTATAACCTTCATGTTGACCTTTAATGATTGTCGTATGCATACTACCATACCTACTGTTTGCATTGCAAATAAGAAGGGTGCAGGTCTTCCTAGTTTTTCAAAGGTTAAGAAATTCTTCTGGTTTTTGTGTCTGTTCATAGTTGCTGTTTTCTTTGCACTAAGCTTTTTTGATTTTAGTACTCAGGTTAGCAGTGATAGTGATTATGACTTCAAGTATATTGAGAGTGGCCAGTTGAAGACTTTTGACAATCCACTTAGTTGTGTGCATAATGTCTTTAGTAACTTCGACCAGTGGCATGATGCCAAGTTTGGTTTCACCCCCGTCAACAATCCTAGTTGTCCTATAGTCGTTGGTGTATCAGACGAAGCGCGCACTGTTCCAGGTATCCCAGCAGGTGTTTATTTAGCTGGTAAAACACTTGTTTTTGCTATTAACACCATTTTTGGTACATCTGGTTTGTGCTTTGATGCTAGTGGCGTTGCTGATAAGGGCGCTTGCATTTTTAATTCGGCTTGCACCACATTATCTGGTTTGGGTGGAACTGCTGTCTACTGTTATAAGAATGGTCTAGTTGAAGGTGCTAAACTTTATAGTGAGTTGGCACCTCATAGCTACTATAAAATGGTAGATGGTAATGCTGTGTCTTTACCTGAAATTATCTCACGCGGCTTTGGCATCCGTACTATCCGTACAAAGGCTATGACCTACTGTCGCGTTGGCCAGTGTGTGCAATCTGCAGAAGGTGTTTGTTTTGGCGCCGATAGATTCTTTGTCTATAATGCAGAATCTGGTTCTGACTTTGTTTGTGGCACAGGGCTCTTTACATTGTTGATGAACGTTATTAGTGTTTTTTCCAAGACAGTACCAGTAACTGTGTTGTCTGGTCAAATACTTTTTAATTGCATTATTGCTTTTGCTGCTGTTGCGGTGTGTTTCTTATTTACAAAGTTTAAGCGCATGTTCGGTGATATGTCTGTTGGCGTTTTCACTGTCGGTGCTTGTACTTTGTTGAACAATGTTTCCTACATTGTAACACAGAACACACTTGGCATGTTGGGCTATGCAACTTTGTACTTTTTGTGCACTAAAGGTGTTAGATATATGTGGATTTGGCATTTGGGATTTTTGATCTCATATATACTTATTGCACCATGGTGGGTTTTGATGGTTTATGCCTTTTCAGCCATTTTTGAGTTTATGCCTAACCTTTTTAAGCTTAAGGTTTCAACACAACTTTTTGAGGGTGACAAGTTCGTAGGCTCTTTTGAAAATGCTGCAGCAGGTACATTTGTGCTTGATATGCATGCCTATGAGAGACTTGCCAACTCTATCTCAACTGAAAAACTGCGTCAGTATGCTAGTACTTACAATAAGTACAAGTATTATTCAGGCAGTGCTTCAGAGGCTGATTACAGGCTTGCTTGTTTTGCCCATTTGGCCAAGGCTATGATGGATTATGCTTCTAATCACAACGACACGTTATACACACCACCCACTGTGAGTTACAATTCAACTCTACAGGCTGGCTTGCGTAAGATGGCACAACCATCTGGTGTTGTTGAGAAGTGCATAGTTCGTGTTTGCTATGGTAATATGGCTCTTAATGGCCTATGGCTTGGTGATACTGTTATCTGCCCACGCCATGTTATAGCGTCTAGTACTACTAGCACTATAGATTATGACTATGCCCTTTCTGTTTTACGCCTCCACAACTTCTCCATTTCATCTGGTAATGTTTTCCTAGGTGTTGTGGGTGTAACCATGCGAGGTGCTTTGTTGCAGATAAAGGTTAATCAAAACAATGTCCACACGCCTAAGTACACCTATCGCACAGTTAGACCGGGTGAATCTTTTAATATCTTGGCGTGCTATGATGGTTCTGCAGCTGGTGTTTACGGCGTTAACATGCGCTCTAATTACACTATTAGAGGCTCGTTCATTAATGGCGCTTGTGGTTCACCTGGTTATAACATTAACAATGGTACCGTTGAGTTTTGCTATTTACACCAGCTTGAACTTGGTTCAGGCTGTCATGTTGGTAGCGACTTAGATGGTGTTATGTATGGTGGTTATGAGGACCAACCTACTTTGCAAGTTGAAGGCGCTAGTAGTCTGTTTACAGAGAATGTGTTGGCATTTCTTTATGCAGCACTCATTAATGGTTCTACCTGGTGGCTTAGTTCTTCTAGGATTGCTGTAGACAGGTTTAATGAGTGGGCTGTTCATAATGGTATGACAACAGTAGTTAATACTGATTGCTTTTCTATTCTTGCTGCTAAGACTGGTGTTGATGTACAACGTTTGTTGGCCTCAATCCAGTCTCTGCATAAGAATTTTGGTGGAAAGCAAATTCTTGGCTATACCTCGTTGACAGATGAGTTTACTACAGGTGAAGTTATACGTCAAATGTATGGCGTTAATCTTCAGAGTGGTTATGTTTCACGCGCCTGTAGAAATGTCTTGCTGGTTGGTTCTTTTCTGACTTTCTTTTGGTCAGAATTAGTTTCCTACACTAAGTTCTTTTGGGTAAATCCTGGTTATGTCACACCTATGTTTGCGTGTTTGTCATTGCTGTCCTCACTTTTGATGTTCACACTCAAGCATAAGACATTGTTTTTCCAGGTCTTTCTAATACCTGCTCTGATTGTTACATCTTGCATTAATTTGGCATTTGATGTTGAAGTCTACAACTATTTGGCAGAGCATTTTGATTACCATGTTTCTCTCATGGGTTTTAATGCACAAGGTCTTGTTAACATCTTTGTCTGCTTTGTTGTTACCATTTTACACGGCACATACACATGGCGCTTTTTTAACACACCTGTGAGTTCTGTCACTTATGTGGTAGCTTTGCTGACTGCGGCATATAACTATTTTTACGCTAGTGACATTCTTAGTTGTGCTATGACACTATTTGCTAGTGTGACTGGCAACTGGTTCGTTGGTGCTGTTTGTTATAAAGCTGCTGTTTATATGGCCTTGAGATTTCCTACTTTTGTGGCTATTTTTGGTGATATTAAGAGTGTTATGTTCTGTTACCTTGTGTTGGGTTATTTTACCTGTTGCTTCTACGGTATTCTCTACTGGTTCAACAGGTTTTTTAAGGTTAGTGTAGGTGTCTATGACTATACTGTTAGTGCTGCTGAGTTTAAGTATATGGTTGCTAACGGCCTACGTGCACCAACTGGAACACTTGATTCACTACTTCTGTCTGCCAAATTGATTGGTATTGGTGGTGAGCGGAATATTAAGATTTCTTCCGTTCAGTCTAAACTGACTGATATTAAGTGTAGTAACGTTGTGCTTTTAGGCTGTCTCTCTAGCATGAATGTCTCAGCAAATTCAACAGAATGGGCCTATTGTGTTGACTTGCATAACAAGATCAACTTGTGTAATGACCCAGAAAAAGCGCAGGAAATGCTACTTGCTTTGTTGGCATTTTTCCTTAGTAAGAATAGTGCTTTTGGTTTAGATGACTTATTGGAATCCTATTTTAATGACAATAGTATGTTGCAGAGTGTTGCATCTACTTATGTCGGTTTGCCTTCTTATGTCATTTATGAAAATGCACGCCAACAGTATGAAGATGCTGTTAATAATGGTTCTCCACCTCAGTTGGTTAAGCAATTGCGCCATGCCATGAATGTAGCAAAGAGCGAATTTGACCGTGAGGCTTCTACTCAGCGTAAGCTTGATAGAATGGCGGAACAGGCTGCAGCACAGATGTACAAAGAGGCACGAGCAGTTAATAGGAAGTCCAAAGTTGTAAGTGCTATGCATTCACTGCTTTTTGGTATGTTGAGACGTTTGGACATGTCTTCTGTAGACACCATTCTCAACTTGGCAAAGGATGGGGTTGTACCTCTGTCTGTCATACCGGCAGTCAGTGCTACTAAGCTTAACATTGTTACTTCTGATATCGATTCTTATAATCGTATCCAGCGTGAGGGATGTGTCCACTACGCTGGTACCATTTGGAATATAATTGATATCAAGGACAATGATGGCAAGGTGGTACACGTTAAGGAGGTAACCGCACAGAATGCTGAGTCCCTGTCATGGCCCCTGGTCCTTGGGTGTGAGCGTATTGTCAAGCTCCAGAATAATGAAATTATTCCCGGTAAGCTGAAGCAGCGCTCCATTAAGGCAGAAGGAGATGGCATAGTTGGAGAAGGTAAGGCACTTTACAATAATGAGGGTGGACGTACTTTTATGTATGCTTTCATCTCGGACAAACCGGACCTGCGTGTAGTCAAGTGGGAGTTCGATGGTGGTTGTAACACTATTGAGCTAGAACCACCACGTAAGTTCTTGGTGGATTCTCCTAATGGTGCACAGATCAAGTATCTCTACTTTGTTCGTAACCTTAACACGTTACGTAGGGGTGCTGTTCTCGGCTACATAGGTGCCACTGTACGCTTGCAGGCTGGTAAACAAACAGAACAGGCTATTAACTCTTCATTGTTGACACTTTGCGCTTTCGCTGTGGATCCTGCTAAGACCTACATCGATGCTGTCAAAAGTGGTCACAAACCAGTAGGTAACTGTGTTAAGATGTTGGCCAATGGTTCTGGTAATGGACAAGCTGTTACTAATGGTGTGGAGGCTAGTACTAACCAGGATTCATACGGTGGTGCGTCCGTGTGTCTATATTGTAGAGCACATGTTGAGCATCCATCTATGGATGGTTTTTGCAGACTGAAAGGCAAGTACGTACAGGTTCCACTAGGTACAGTGGATCCTATACGTTTTGTACTTGAGAATGACGTTTGCAAGGTTTGTGGTTGTTGGCTGGCTAATGGCTGCACTTGTGACAGATCCATTATGCAAAGCACTGATATGGCTTATTTAAACGAGTACGGGGCTCTAGTGCAGCTCGACTAGAGCCCTGTAACGGTACTGATACACAACATGTGTATCGTGCTTTTGACATCTACAACAAGGATGTTGCTTGTCTAGGTAAATTCCTCAAGGTGAACTGTGTTCGCCTGAAGAATTTGGATAAGCATGATGCATTCTATGTTGTCAAAAGATGTACCAAGTCTGCGATGGAACACGAGCAATCCATCTATAGCAGACTTGAAAAGTGTGGAGCCGTAGCCGAACACGATTTCTTCACTTGGAAGGATGGTCGTGCCATCTATGGTAACGTTTGTAGAAAGGATCTTACCGAGTATACTATGATGGATTTGTGTTACGCTTTACGTAACTTTGATGAAAACAATTGCGATGTTCTTAAGAGCATTTTAATTAAGGTAGGCGCTTGTGAGGAGTCCTACTTCAATAATAAAGTCTGGTTTGACCCTGTTGAAAATGAAGACATTCATCGTGTCTATGCATTGTTAGGTACCATTGTTTCACGTGCTATGCTTAAATGCGTTAAGTTCTGTGATGCAATGGTTGAACAAGGTATAGTTGGTGTTGTCACATTAGATAATCAGGATCTTAATGGTGATTTTTATGATTTTGGTGATTTTACTTGTAGCATCAAGGGAATGGGTATACCCATTTGCACATCATATTACTCTTATATGATGCCTGTTATGGGTATGACTAATTGCCTTGCTAGTGAGTGTTTTGTTAAGAGTGATATATTTGGTGAGGATTTCAAGTCATATGACCTGCTGGAATATGATTTCACGGAGCATAAGACAGCACTCTTCAACAAGTATTTCAAGTATTGGGGACTGCAATACCACCCTAACTGTGTGGACTGCAGTGATGAGCAGTGCATAGTTCACTGTGCCAACTTCAATACGTTGTTTTCCACTACTATACCTATTACGGCATTTGGACCTTTGTGTCGCAAGTGTTGGATTGATGGTGTTCCACTGGTAACTACAGCTGGTTATCATTTTAAACAGTTAGGTATAGTTTGGAACAATGACCTCAACTTACACTCTAGCAGGCTCTCTATTAACGAATTACTCCAGTTTTGTAGTGATCCTGCATTGCTTATAGCATCATCACCAGCCCTTGTTGATCAGCGTACTGTTTGCTTTTCAGTTGCAGCGCTAGGTACAGGTATGACTAACCAGACTGTTAAACCTGGCCATTTCAATAAGGAGTTTTATGACTTCTTACTTGAGCAAGGTTTCTTTTCTGAGGGCTCTGAGCTTACTTTAAAGCACTTCTTCTTTGCACAGAAGGGTGATGCAGCTGTTAAGGATTTTGACTACTATAGGTATAATAGACCTACTGTTCTGGACATTTGCCAAGCTCGCGTCGTGTATCAAATAGTGCAACGCTATTTTGATATTTACGAAGGTGGTTGTATCACTGCTAAAGAGGTGGTTGTTACAAACCTTAACAAGAGCGCAGGTTATCCTTTGAACAAGTTTGGTAAAGCTGGTCTTTACTATGAGTCTTTATCCTATGAGGAACAGGATGAACTTTATGCTTATACTAAGCGTAACATCCTGCCCACTATGACACAGCTCAACCTTAAATATGCTATAAGTGGCAAAGAACGTGCACGCACAGTGGGTGGTGTTTCGCTTTTGTCAACCATGACTACTCGGCAGTATCATCAGAAACACCTTAAGTCCATAGTTAATACTAGGGGCGCTTCGGTTGTTATTGGTACTACTAAGTTTTATGGTGGTTGGGACAATATGCTTAAGAACCTTATTGATGGTGTTGAAAATCCGTGTCTTATGGGTTGGGACTACCCAAAGTGCGACAGAGCACTGCCCAATATGATACGTATGATTTCAGCCATGATTTTAGGCTCTAAGCACACCACATGCTGCAGTTCCACTGACCGCTTTTTCAGGTTGTGCAATGAATTGGCTCAAGTCCTTACTGAGGTTGTTTATTCTAATGGAGGTTTTTATTTGAAGCCAGGTGGTACTACCTCTGGTGATGCAACCACCGCATATGCAAACTCAGTTTTTAATATCTTCCAAGCAGTAAGTGCCAATGTTAACAAACTTCTTAGTGTTGACAGCAATGTCTGTCATAATTTAGAAGTTAAGCAATTGCAGCGTAAGCTTTATGAGTGCTGTTATAGATCAACTACCGTCGATGACCAGTTCGTCGTTGAGTATTATGGTTACTTGCGTAAACATTTTTCAATGATGATTCTTTCTGATGATGGCGTTGTTTGTTATAACAATGACTATGCATCACTTGGTTATGTCGCTGATCTTAACGCATTCAAGGCTGTTTTGTATTACCAGAACAATGTCTTCATGAGCGCCTCTAAATGTTGGATCGAGCCTGACATTAATAAAGGTCCTCATGAATTTTGCTCGCAGCATACTATGCAGATTGTCGATAAAGATGGTACTTATTACCTTCCTTACCCTGATCCTTCAAGAATTCTCTCTGCAGGTGTGTTTGTTGATGACGTTGTTAAAACTGATGCAGTTGTATTGCTTGAACGTTATGTGTCATTGGCTATAGATGCCTACCCGTTATCTAAGCATGAAAACCCTGAATATAAGAAGGTGTTTTATGTGCTTTTGGATTGGGTTAAGCATCTGTACAAAACTCTTAATGCTGGTGTGTTAGAGTCTTTTTCTGTCACACTTTTGGAAGATTCTACTGCTAAATTCTGGGATGAGAGCTTTTATGCCAACATGTATGAGAAATCTGCAGTTTTACAATCTGCAGGGCTTTGTGTTGTTTGTGGCTCTCAAACTGTTTTACGTTGTGGTGATTGTCTACGGCGTCCTATGCTTTGTACTAAGTGTGCTTATGATCATGTCATTGGAACAACTCACAAGTTCATTTTGGCCATCACTCCATATGTGTGTTGTGCTTCAGATTGTGGTGTCAATGATGTAACTAAGCTCTACTTAGGTGGTCTTAGTTATTGGTGTCATGACCACAAGCCACGTCTTGCATTCCCGTTGTGCTCTGCTGGTAATGTTTTTGGCTTGTACAAAAATTCTGCTACCGGCTCACCCGATGTTGAAGACTTTAATCGCATTGCTACATCCGATTGGACTGATGTTTCTGACTACAGGTTGGCAAATGATGTCAAGGACTCATTGCGTCTGTTTGCAGCGGAAACTATCAAGGCCAAGGAGGAGAGCGTTAAGTCATCCTATGCTTGTGCAACACTACATGAGGTTGTAGGACCTAAAGAGTTGTTGCTCAAATGGGAAGTCGGCAGACCCAAACCACCCCTTAATAGAAATTCGGTTTTCACTTGTTATCATATAACGAAGAACACCAAATTTCAAATCGGTGAGTTTGTGTTTGAGAAGGCAGAATATGATAATGATGCTGTAACATATAAAACTACCGCCACAACAAAACTTGTTCCTGGCATGGTTTTTGTGCTTACCTCACATAATGTTCAGCCATTGCGCGCACCGACCATTGCTAATCAAGAACGTTATTCCACTATACATAAGTTGCATCCTGCTTTTAACATACCTGAAGCTTATTCTAGCTTAGTGCCCTATTACCAATTGATTGGTAAGCAGAAGATTACAACTATTCAGGGACCTCCCGGTAGTGGTAAATCTCACTGTGTTATAGGGCTAGGTTTGTACTATCCAGGTGCACGTATAGTGTTTACAGCTTGTTCTCATGCAGCGGTCGATTCACTTTGTGTGAAAGCTTCCACTGCTTATAGCAATGACAAATGTTCACGCATCATACCACAGCGCGCTCGTGTTGAGTGTTATGATGGTTTCAAGTCTAATAATACTAGTGCTCAGTACCTTTTCTCTACTGTCAATGCTTTGCCAGAGTGCAATGCGGACATTGTTGTGGTGGATGAGGTCTCTATGTGCACTAATTATGACTTGTCTGTCATAAATCAGCGCATCAGCTATAGGCATGTAGTCTATGTTGGTGACCCTCAACAGCTGCCTGCACCACGTGTTATGATTTCACGTGGTACTTTGGAACCAAAGGACTACAACGTTGTCACTCAACGCATGTGTGCCCTTAAGCCTGATGTTTTCTTGCACAAGTGTTATCGCTGTCCTGCTGAGATAGTGCGTACTGTGTCTGAGATGGTCTATGAAAACCAATTCATTCCTGTGCACCCAGATAGCAAGCAGTGTTTTAAAATCTTTTGCAAGGGTAATGTTCAGGTTGATAATGGTTCAAGCATTAATCGCAGGCAATTGGATGTTGTGCGTATGTTTTTGGCTAAAAATCCTAGGTGGTCAAAGGCTGTTTTTATTTCTCCTTATAACAGCCAGAATTATGTTGCCAGCCGCATGCTAGGTCTACAAATTCAGACAGTTGACTCATCCCAGGGTAGTGAGTATGACTATGTCATTTACACACAAACTTCAGATACTGCCCATGCCTGTAATGTTAACAGGTTTAATGTTGCCATCACAAGGGCCAAGAAAGGCATATTATGTATAATGTGCGATAGGTCCCTTTTTGATGTGCTTAAATTCTTTGAGCTTAAATTGTCTGATTTGCAGGCTAATGAGGGTTGTGGTCTTTTTAAAGACTGTAGCAGAGGTGATGATCTGTTGCCACCATCTCACGCTAACACCTTCATGTCTTTAGCGGACAATTTTAAGACTGATCAAGATCTTGCTGTTCAAATAGGTGTTAATGGACCCATTAAATATGAGCATGTTATCTCGTTTATGGGTTTCCGTTTTGATATCAACATACCCAACCATCATACTCTCTTTTGCACACGCGACTTTGCCATGCGCAATGTTAGAGGTTGGTTAGGCTTTGACGTTGAAGGAGCACATGTTGTTGGCTCTAACGTCGGTACAAATGTCCCATTGCAATTAGGGTTTTCTAACGGTGTTGATTTTGTTGTCAGACCTGAAGGTTGCGTTGTAACAGAGTCTGGTGACTACATTAAACCCGTCAGAGCTCGTGCTCCACCAGGGGAACAATTCGCACACCTTTTGCCTTTACTTAAACGCGGCCAACCATGGGATGTTGTCCGCAAACGTATAGTGCAGATGTGTAGTGACTACCTGGCCAACCTATCAGACATACTAATTTTTGTGTTGTGGGCTGGTGGTTTGGAGTTGACAACTATGCGTTATTTTGTCAAGATTGGACCAAGTAAGAGTTGTGATTGTGGTAAGGTTGCTACTTGTTACAATAGTGCGCTGCATACGTACTGTTGTTTCAAACATGCCCTTGGTTGTGATTATCTGTATAACCCATACTGTATTGATATACAGCAGTGGGGATACAAGGGATCACTTAGCCTTAACCACCATGAGCATTGTAATGTACATAGAAACGAGCATGTGGCTTCTGGTGATGCCATAATGACTCGCTGTCTGGCCATACATGATTGCTTTGTCAAGAACGTTGACTGGTCCATCACATACCCATTTATTGGTAATGAGGCTGTTATTAATAAGAGCGGCCGAATTGTGCAATCACACACTATGCGGTCAGTTCTTAAGTTATACAATCCGAAAGCCATATATGATATTGGCAATCCTAAGGGCATTAGATGTGCCGTAACGGATGCTAAGTGGTTTTGCTTTGACAAGAATCCTACTAATTCTAATGTCAAGACATTGGAGTATGACTATATAACACATGGCCAATTTGATGGGTTGTGCTTGTTTTGGAATTGCAATGTAGACATGTATCCAGAATTTTCTGTGGTCTGTCGTTTTGATACTCGCTGTAGGTCACCACTCAACTTGGAGGGTTGTAATGGTGGTTCACTGTATGTTAATAATCATGCATTCCATACACCGGCTTTTGACAAGCGTGCTTTTGCTAAGTTGAAGCCAATGCCATTTTTCTTTTATGATGATACTGAGTGTGACAAGTTACAGGACTCCATAAACTATGTTCCTCTTAGGGCTAGTAACTGCATTACTAAATGTAATGTTGGTGGTGCTGTCTGTAGTAAGCATTGTGCTATGTATCATAGCTATGTTAATGCTTACAACACTTTTACGTCGGCGGGCTTTACTATTTGGGTGCCTACTTCGTTTGACACCTATAATCTGTGGCAGACATTTAGTAACAATTTGCAAGGTCTTGAGAACATTGCTTTCAATGTCGTAAAGAAAGGATCTTTTGTTGGTGCCGAAGGTGAACTTCCTGTAGCTGTGGTTAATGACAAAGTGCTCGTTAGAGATGGTACTGTTGATACTCTTGTTTTTACAAACAAGACATCACTACCCACTAACGTAGCTTTTGAGTTGTATGCCAAGCGTAAGGTAGGACTCACCCCACCCATTACGATCCTACGTAACTTGGGTGTAGTTTGCACATCTAAGTGTGTCATTTGGGACTATGAAGCCGAACGTCCACTTACTACTTTTACAAAGGATGTTTGTAAATATACCGACTTTGAGGGTGACGTCTGTACACTCTTTGATAACAGCATTGTTGGTTCATTAGAGCGATTCTCCATGACCCAAAATGCTGTGCTTATGTCACTTACAGCTGTTAAAAAGCTTACTGGCATAAAGTTAACTTATGGTTATCTTAATGGTGTCCCAGTTAACACACATGAAGATAAACCTTTTACTTGGTATATTTACACTAGGAAGAACGGCAAGTTCGAGGACCATCCTGATGGCTATTTTACCCAAGGTAGAACAACCGCTGATTTTAGCCCTCGTAGCGACATGGAAAAGGACTTCCTAAGTATGGATATGGGTCTGTTTATTAACAAGTACGGACTTGAAGATTACGGCTTTGAGCACGTTGTGTATGGTGATGTTTCAAAAACCACCCTTGGTGGTTTGCATCTACTAATTTCGCAGGTGCGTCTGGCCTGTATGGGTGTGCTCAAAATAGACGAGTTTGTGTCTAGTAATGATAGCACGTTAAAGTCTTGTACTGTTACATATGCTGATAACCCTAGTAGTAAGATGGTTTGTACGTATATGGATCTCCTGCTTGACGATTTTGTCAGCATTCTTAAATCTTTGGATTTGGGCGTTGTATCTAAAGTTCATGAAGTTATGGTCGATTGTAAAATGTGGAGGTGGATGTTGTGGTGTAAGGATCATAAACTCCAGACATTTTATCCGCAACTTCAGGCCAGTGAATGGAAGTGTGGTTATTCCATGCCTTCTATTTACAAGATACAACGTATGTGTTTAGAACCTTGCAATCTCTACAACTATGGTGCTGGTATTAAGTTACCTGATGGCATTATGTTTAACGTAGTTAAATACACACAGCTTTGTCAATATCTCAATAGCACCACAATGTGTGTACCCCATCACATGCGTGTGCTACATCTTGGTGCTGGCTCCGACAAGGGTGTTGCACCTGGCACGGCTGTCTTACGACGTTGGTTGCCACTGGATGCCATTATAGTTGACAATGATAGTGTGGATTACGTTAGCGATGCTGATTATAGTGTTACAGGAGATTGCTCTACCTTATACCTGTCAGATAAGTTTGATTTAGTTATATCTGATATGTATGATGGTAAGATTAAAAGTTGTGATGGGGAGAACGTGTCTAAAGAAGGCTTCTTTCCCTATATTAATGGTGTCATCACCGAAAAGTTGGCACTTGGTGGTACTGTAGCTATTAAGGTGACGGAGTTTAGTTGGAATAAGAAGTTGTATGAACTCATTCAGAGGTTTGAGTATTGGACAATGTTCTGTACCAGTGTTAACACGTCATCGTCAGAGGCATTCTTAATTGGTGTTCACTATTTAGGTGATTTTGCAAGTGGCGCTGTGATTGACGGCAACACTATGCATGCCAATTATATCTTCTGGCGTAATTCCACAATTATGACTATGTCTTACAATAGTGTACTTGATTTAAGCAAGTTCAATTGTAAGCATAAGGCTACAGTTGTCATTAATTTAAAAGATTCATCCATTAGTGATGTTGTGTTAGGTTTGTTGAAGAATGGTAAGTTGCTAGTGCGTAATAATGACGCCATTTGTGGTTTTTCTAATCATTTGGTCAACGTAAACAAATGAAGTCTTTAACCTACTTCTGGTTGTTCTTACCAGTACTTTCAACACTTAGCCTACCACAAGATGTCACCAGGTGCTCAGCTAACACTAATTTTAGGCGGTTCTTTTCAAAATTTAATGTTCAGGCGCCTGCAGTTGTTGTACTGGGCGGTTATCTACCTAAAAACCAGGGTGTCAATTCAACTTGGTACTGTGCTGGCCAACATCCAACTGCTAGTGGCGTTCATGGTATCTTTGTTAGCCATATTAGAGGTGGTCATGGCTTTGAGATTGGCATTTCGCAAGAGCCTTTTGACCCTAGTGGTTACCAGCTTTATTTACATAAGGCTACTAACGGTAACACTAATGCTACTGCGCGACTGCGCATTTGCCAGTTTCCTAGCATTAAAACATTGGGCCCCACTGCTAATAATGATGTTACAACAGGTCGTAATTGCCTATTTAACAAAGCCATCCCAGCTCATATGAGTGAACATAGTGTTGTCGGCATAACATGGGATAATGATCGTGTCACTGTCTTTTCTGACAAGATCTATTATTTTTATTTTAAAAATGATTGGTCCCGTGTTGCGACAAAGTGTTACAACAGTGGAGGTTGTGCTATGCAATATGTTTACGAACCCACCTATTACATGCTTAATGTTACTAGTGCTGGTGAGGATGGTATTTCTTACCAACCCTGTACAGCTAATTGCATTGGTTATGCTGCCAATGTATTTGCTACTGAGCCCAATGGCCACATACCAGAAGGTTTTAGTTTTAATAATTGGTTTCTTTTGTCCAATGATTCCACTTTGGTGCATGGTAAGGTGGTTTCCAACCAACCATTGTTGGTCAATTGTCTTTTGGCCATTCCTAAGATTTATGGACTAGGCCAATTTTTCTCCTTTAATCAAACGATCGATGGTGTTTGTAATGGAGCTGCTGTGCAGCGTGCACCAGAGGCTCTGAGGTTTAATATTAATGACATCTCTGTCATTCTTGCTGAAGGCTCAATTGTACTTCATACTGCTTTAGGAACAAATTTTTCTTTTGTTTGCAGTAATTCCTCAAATCCTCACTTAAAGAAGAAAGGTGCCACCTTCGCCATACCTCTGGGTGCTACCCAAGTACCTTATTATTGTTTTTTTAAAGTGGATACTTACAACTCCACTGTTTATAAATTTTTGGCTGTTTTACCTCCTACCGTCAGGGAAATTGTCATCACCAAGTATGGTGATGTTTATGTCAATGGGTTTGGATACTTGCATCTCGGTTTGTTGGATGCTGTCACAATTAATTTCACTGGTCATGGCACTGACGATGATGTTTCTGGTTTTTGGACCATAGCATCGACTAATTTTGTTGATGCACTCATCGAAGTTCAAGGAACCGCCATTCAGCGTATTCTTTATTGTGATGATCCTGTTAGCCAACTCAAGTGTTCTCAGGTTGCTTTTGACCTTGACGATGGTTTTTACACTATTTCTTCTAGAAACCTTCTGAGTCATGAACAGCCAATTTCTTTTGTTACTCTGCCATCATTTAATGATCATTCTTTTGTTAACATTACTGTATCTGCTTCCTTTGGTGGTCATAGTGGTGCCAACCTTATTGCATCTGACACTACTATCAATGGGTTTAGTTCTTTCTGTGTTGACACTAGACAATTTACCATTTCACTGTTTTATAACGTTACAAACAGTTATGGTTATGTGTCTAAATCACAGGACAGTAATTGCCCTTTCACCTTGCAATCTGTTAATGATTACCTGTCTTTTAGCAAATTTTGTGTTTCCACCAGCCTTTTGGCTAGTGCCTGTACCATAGATCTTTTTGGTTACCCTGAGTTTGGTAGTGGTGTTAAGTTTACGTCCCTTTACTTTCAATTCACAAAGGGTGAGTTGATTACTGGCACGCCTAAACCACTTGAAGGTGTCACGGACGTTTCTTTTATGACTCTGGATGTGTGTACCAAGTATACTATCTATGGCTTTAAAGGTGAGGGTATCATTACCCTTACAAATTCTAGCTTTTTGGCAGGTGTTTATTACACATCTGATTCTGGACAGTTGTTAGCCTTTAAGAATGTCACTAGTGGTGCTGTTTATTCTGTTACGCCATGTTCTTTTTCAGAGCAGGCTGCATATGTTGATGATGATATAGTGGGTGTTATTTCTAGTTTGTCTAGCTCCACTTTTAACAGTACTAGGGAGTTGCCTGGTTTCTTCTACCATTCTAATGATGGCTCTAATTGTACAGAGCCTGTGTTGGTGTATAGTAACATAGGTGTTTGTAAATCTGGCAGTATTGGCTACGTCCCATCTCAGTCTGGCCAAGTCAAGATTGCACCCACGGTTACTGGGAATATTAGTATTCCCACCAACTTTAGTATGAGTATTAGGACAGAATATTTACAGCTTTACAACACGCCTGTTAGTGTTGATTGTGCCACATATGTTTGTAATGGTAACTCTCGTTGTAAACAATTACTCACCCAGTACACTGCAGCATGTAAGACCATAGAGTCAGCATTACAACTCAGCGCTAGGCTTGAGTCTGTTGAAGTTAACTCTATGCTTACTATTTCTGATGAGGCTCTACAGTTAGCTACCATTAGTTCGTTTAATGGTGATGGATATAATTTTACTAATGTGCTGGGTGTTTCTGTGTATGATCCTGCAAGTCGCAGGGTGGTACAAAAAAGGTCTTTTATTGAAGACCTGCTTTTTAATAAAGTGGTTACTAATGGCCTTGGTACTGTTGATGAAGACTATAAGCGCTGTTCTAATGGTCGCTCTGTGGCAGATCTAGTCTGTGCACAGTATTACTCTGGTGTCATGGTACTACCTGGTGTTGTTGACGCTGAGAAGCTTCACATGTATAGTGCGTCTCTCATCGGTGGTATGGTGCTAGGAGGTTTTACTTCTGCAGCGGCATTGCCTTTTAGCTATGCTGTTCAAGCTAGACTCAATTATCTTGCTCTACAGACGGATGTTCTACAGCGGAACCAGCAATTGCTTGCTGAGTCTTTTAACTCTGCTATTGGTAATATAACTTCAGCCTTTGAGAGTGTTAAAGAGGCTATTAGTCAAACTTCCAAGGGTTTGAACACTGTGGCTCATGCGCTTACTAAGGTTCAAGAGGTTGTTAACTCGCAGGGTGCAGCTTTGACTCAACTTACCGTACAGCTGCAACACAACTTCCAAGCCATTTCTAGTTCTATTGATGACATTTACTCTCGACTGGACATTCTTTCAGCCGATGCTCAGGTTGACCGTCTCATCACCGGCAGATTATCAGCACTTAATGCTTTTGTTGCTCAAACCCTCACTAAGTATACTGAGGTTCAGGCTAGCAGGAAGTTAGCACAGCAAAAGGTTAATGAGTGCGTTAAATCGCAATCTCAGCGTTATGGTTTTTGTGGTGGTGATGGCGAGCACATTTTCTCTCTGGTACAGGCAGCACCTCAGGGCCTGCTGTTTTTACATACAGTACTTGTACCGAGTGATTTTGTAGATGTTATTGCCATCGCTGGCTTATGCGTTAACGATGAAATTGCCTTGACTCTACGTGAGCCTGGCTTAGTCTTGTTTACGCATGAACTTCAAAATCATACTGCGACGGAATATTTTGTTTCATCGCGACGTATGTTTGAACCTAGAAAACCTACCGTTAGTGATTTTGTTCAAATTGAGAGTTGTGTGGTCACCTATGTCAATTTGACTAGAGACCAACTACCAGATGTAATCCCAGATTACATCGATGTTAACAAAACACTTTATGAGATTTTAGCTTCTCTGCCCAATAGAACTGGTCCAAGTCTTCCTTTAGATGTTTTTAATGCCACTTATCTTAATCTCACTGGTGAAATTGCAGATTTAGAGCAGCGTTCAGAGTCTCTCCGTAATACTACAGAGGAGCTCCAAAGTCTTATATATAATATCAACAACACACTAGTTGACCTTGAGTGGCTCAACCGAGTTGAGACATATATCAAGTGGCCGTGGTGGGTTTGGTTGATTATTTTCATTGTTCTCATCTTTGTTGTGTCATTACTAGTGTTCTGCTGCATTTCCACGGGTTGTTGTGGATGCTGCGGCTGCTGCTGTGCTTGTTTCTCAGGTTGTTGTAGGGGTCCTAGACTTCAACCTTACGAAGTTTTTGAAAAGGTCCACGTGCATTGATGTTTCTTGGACTTTTTCAATACACGATTGACACAGTTGTCAAAGATGTCTCAAAGTCTGCTAACTTGTCTTTGGATGCTGTCCAAGAGTTGGAGCTCAATGTAGTTCCAATTAGACAAGCTTCAAATGTGACGGGTTTTCTTTTCACCAGTGTTTTTATCTACTTCTTTGCACTGTTTAAAGCGTCTTCTTTGAGGCGCAATTATATTATGTTGGCAGCGCGTTTTGCTGTCATTGTTCTTTATTGCCCACTTTTATATTATTGTGGTGCATTTTTAGATGCAACTATTATTTGTTGCACACTTATTGGCAGGCTTTGTTTAGTCTGCTTTTACTCCTGGCGCTATAAAAATGCGCTCTTTATTATTTTTAATACTACGACACTTTCTTTCCTCAATGGTAAAGCAGCTTATTATGACGGCAAATCCATTGTGATTTTAGAAGGTGGTGACCATTACATCACTTTTGGCAACTCTTTTGTTGCTTTTGTTAGTAGCATCGACTTGTATCTAGCTATACGTGGGCGGCAAGAAGCTGACCTACAGCTGTTGCGAACTGTTGAGCTTCTTGATGGCAAGAAGCTTTATGTCTTTTCGCAACATCAAATTGTTGGCATTACTAATGCTGCATTTGACTCAATTCAACTAGACGAGTATGCTACAATTAGTGAATGATAATGGTCTAGTAGTTAATGTTATACTTTGGCTTTTCGTACTCTTTTTCCTGCTTATTATAAGCATTACTTTCGTCCAATTGGTTAATCTGTGCTTCACTTGTCACCGGTTGTGTAATAGCGCAGTTTACACACCTATAGGGCGTTTGTATAGAGTTTATAAGTTTTACATGCAAATAGACCCCCTCCCTAGTACTGTTATTGACGTATAAACGAAATATGTCTAACGGTTCTATTCCCGTTGATGAGGTGATTCAACACCTTAGAAACTGGAATTTCACATGGAATATCATACTGACGATACTACTTGTAGTGCTTCAGTATGGCCATTACAAGTACTCTGCGTTCTTGTATGGTGTCAAGATGGCTATTCTATGGATACTTTGGCCTCTTGTGTTAGCACTGTCACTTTTTGATGCATGGGCTAGCTTTCAGGTCAATTGGGTCTTTTTTGCTTTCAGCATCCTTATGGCTTGCATCACTCTTATGCTGTGGATAATGTACTTTGTCAATAGCATTCGGTTGTGGCGCAGGACACATTCTTGGTGGTCTTTCAATCCTGAAACAGACGCGCTTCTCACTACTTCTGTGATGGGCCGACAGGTCTGCATTCCAGTGCTTGGAGCACCAACTGGTGTAACGCTAACACTCCTTAGTGGTACATTGCTTGTAGAGGGCTATAAGGTTGCTACTGGCGTACAGGTAAGTCAATTACCTAATTTCGTCACAGTCGCCAAGGCCACTACAACAATTGTCTACGGACGTGTTGGTCGTTCAGTCAATGCTTCATCTGGCACTGGTTGGGCTTTCTATGTCCGGTCCAAACACGGCGACTACTCAGCTGTGAGTAATCCGAGTTCGGTTCTCACAGATAGTGAGAAAGTGCTTCATTTAGTCTAAACAGAAACTTTATGGCTTCTGTCAGTTTTCAGGATCGTGGCCGCAAACGGGTGCCATTATCCCTCTATGCCCCTCTTAGGGTTACTAATGACAAACCCCTTTCTAAGGTACTTGCAAATAATGCTGTACCCACTAATAAAGGAAATAAGGACCAGCAAATTGGATACTGGAATGAGCAAATTCGCTGGCGCATGCGCCGTGGTGAGCGAATTGAACAACCTTCCAATTGGCATTTCTACTACCTCGGAACAGGACCTCACGCCGACCTCCGCTATAGGACTCGTACTGAGGGTGTTTTCTGGGTTGCTAAAGAAGGCGCAAAGACTGAACCCACTAACCTGGGTGTCAGAAAGGCGTCTGAAAAGCCAATTATTCCAAATTTCTCTCAACAGCTTCCCAGCGTAGTTGAGATTGTTGAACCTAACACACCTCCTACTTCACGTGCAAATTCACGTAGCAGGAGTCGTGGTAATGGCAACAACAGGTCCAGATCTCCAAGTAACAACAGAGGCAATAACCAGTCCCGCGGTAATTCACAGAATCGTGGAAATAACCAGGGTCGTGGAGCTTCTCAGAACAGAGGAGGCAATAATAATAACAATAACAAGTCTCGTAACCAGTCCAAGAACAGAAACCAGTCAAATGACCGTGGTGGTGTAACATCACGCGATGATCTGGTGGCTGCTGTCAAGGATGCCCTTAAATCTTTGGGTATTGGCGAAAACCCTGACAAGCTTAAGCAACAGCAGAAGCCCAAACAGGAAAGGTCTGACAGCAGCGGCAAAAATACACCTAAGAAGAACAAATCCAGAGCCACTTCGAAAGAACGTGACCTCAAAGACATCCCAGAGTGGAGGAGAATTCCCAAGGGCGAAAATAGCGTAGCAGCTTGCTTCGGACCCAGGGGAGGCTTCAAAAATTTTGGAGATGCGGAATTTGTCGAAAAAGGTGTTGATGCCTCAGGCTATGCTCAGATCGCCAGTTTAGCACCAAATGTTGCAGCATTGCTCTTTGGTGGTAATGTGGCTGTTCGTGAGCTAGCGGACTCTTACGAGATTACATATAATTATAAAATGACTGTGCCAAAGTCTGATCCAAATGTAGAGCTTCTTGTTTCACAGGTGGATGCATTTAAAACTGGGAATGCAAAACCCCAGAGAAAGAAGGAAAAGAAGAACAAGCGTGAAACCACGCAGCAGCTGAATGAAGAGGCCATCTACGATGATGTGGGTGTGCCATCTGATGTGACTCATGCCAATTTGGAATGGGACACAGCTGTTGATGGTGGTGACACGGCCGTTGAAATTATCAACGAGATCTTCGACACAGGAAATTAAACAATGTTTGACTGGCTTATCCTGGCTATGTCCCAGGGTAGTGCCATTACACTGTTATTACTGAGTGTTTTTCTAGCGACTTGGCTGCTGGGCTATGGCTTTGCCCTCTAACTAGCGGTCTTGGTCTTGCACACAACGGTAAGCCAGTGGTAATGTCAGTGCAAGAAGGATATTACCATAGCACTGTCATGAGGGGAACGCAGTACCTTTTCATCTAAACCTTTGCACGAGTAATCAAAGATCCGCTTGACGAGCCTATATGGAAGAGCGTGCCAGGTATTTGACTCAAGGACTGTTAGTAACTGAAGACCTGACGGTGTTGATATGGATACAC

//


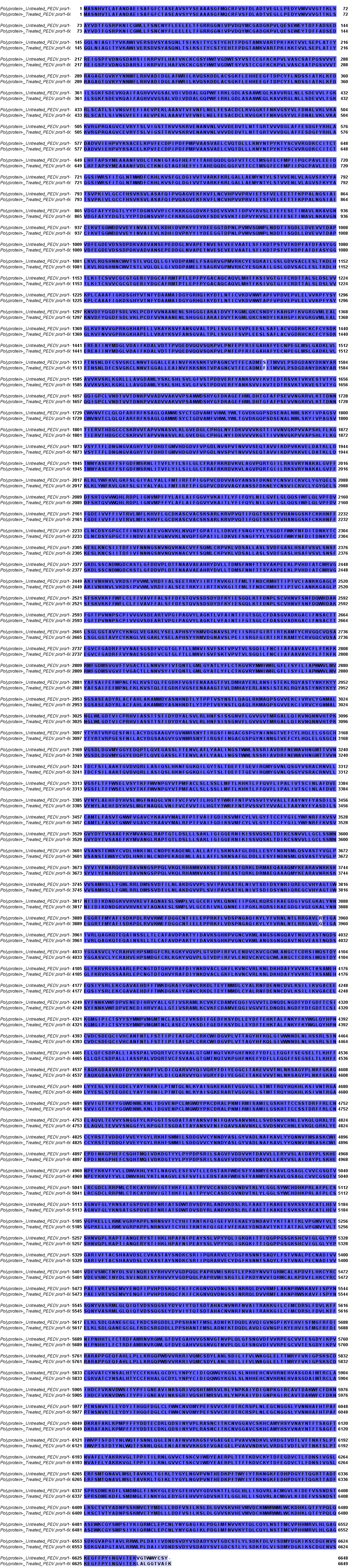


**Supplementary Fig 1: Amino acid sequence alignment for the polyprotein:**

**
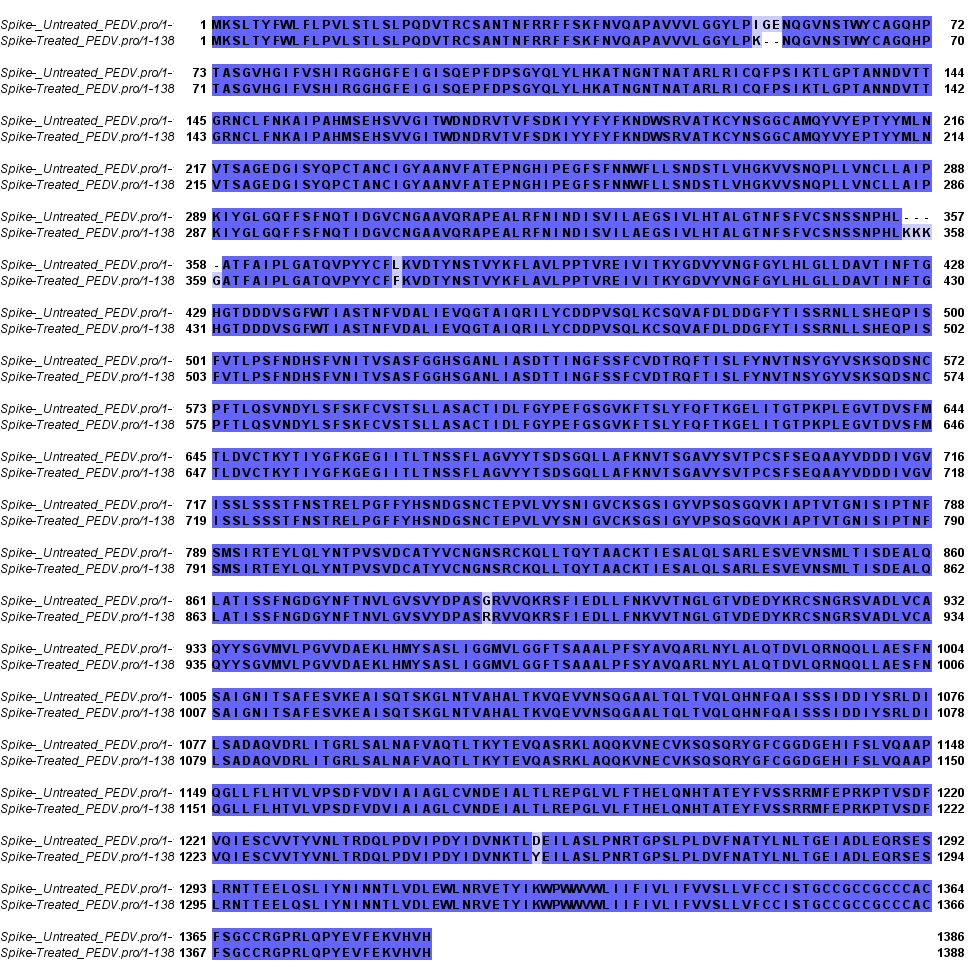
**

**Supplementary Fig 2:** **Amino acid sequence alignment for the spike protein:**


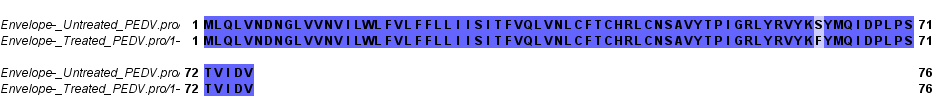


**Supplementary Figure 3**.  **Amino acid sequence alignment for the envelope protein**

**Supporting information legends:**

**Supplementary Fig 1. Amino acid sequence alignment for the polyprotein:** Consensus sequences of the polyproteins of untreated and heat and RNAse treated PEDV virions aligned using the Jalview 2.4 software. Mismatches are indicated by a light blue shading and gaps by hyphens.

**Supplementary Fig 2. Amino acid sequence alignment for the spike protein:** Consensus sequences of the spike proteins of untreated and heat and RNAse treated PEDV virions aligned using the Jalview 2.4 software. Mismatches are indicated by a light blue shading and gaps by hyphens.

**Supplementary Fig 3. Amino acid sequence alignment for the envelope protein:** Consensus sequences of the spike proteins of untreated and heat and RNAse treated PEDV virions aligned using the Jalview 2.4 software. Mismatches are indicated by a light blue shading and gaps by hyphens.
